# Challenges of healthcare financing in the world’s largest refugee camp: a mixed-method study among healthcare stakeholders for Rohingya refugees in Bangladesh

**DOI:** 10.1136/bmjopen-2023-083021

**Published:** 2025-01-23

**Authors:** Imdadul Haque Talukdar, Sayeeda Tarannum, Isabel Smith, M A Rifat, Priyanka Boga, Jubayer Mumin, Pablo André Veronés, Ziba Mahdi, Syeda Saima Alam, Arif Faysal Khan

**Affiliations:** 1 Department of Learning, Informatics, Management, and Ethics, Karolinska Institute, Stockholm, Sweden; 2 Ministry of Public Administration, People's Republic of Bangladesh, Dhaka, Bangladesh; 3 Department of Global Public Health, Karolinska Institutet, Stockholm, Sweden; 4 Department of Economics, North South University, Dhaka, Bangladesh; 5 Department of Food Technology and Nutrition Science, Noakhali Science and Technology University, Noakhali, Bangladesh

**Keywords:** Refugees, HEALTH ECONOMICS, Health policy, PUBLIC HEALTH

## Abstract

**Abstract:**

**Objectives:**

This study aimed to increase the understanding of healthcare stakeholders’ viewpoints on the challenges and potential solutions regarding healthcare financing for the Rohingya refugees in Cox’s Bazar.

**Design:**

A mixed-method approach, containing semi-structured interviews with healthcare stakeholders and review of financial documents, was employed. Thematic analysis was performed to analyse the transcripts. Financial documents (available online) and reports from respective coordinating agencies were also reviewed.

**Setting:**

Online key informant interviews (KIIs) were conducted from Stockholm with participants residing in Bangladesh between June 2022 and September 2022.

**Participants:**

Eight KIIs were conducted with professional health programme managers, executives and personnel involved in policy coordination and implementation in refugee camps in Cox’s Bazar district of Bangladesh.

**Results:**

We identified four themes and three subthemes outlining key challenges in healthcare funding, including decreasing funds (supported by a quantitative assessment) due to macroglobal issues, conflicting short-term and long-term priorities between implementing partners, insufficient efficacy due to challenges with collaborative priority-setting and implementing common processes, and a lack of consensus regarding equity between host and refugee communities.

**Conclusions:**

The study identified unique challenges beyond the commonly discussed health financing issues in a resource-deficient setting: stakeholders’ conflicting priorities regarding funding and undecided equity issues among the host and refugee communities are worth scholarly and policy focus. A needs-based, equitable and effective action plan might ensure the proper utilisation of resources.

**STRENGTHS AND LIMITATIONS OF THIS STUDY**

- Bangladeshi and international researchers collaborated to deepen the study’s understanding of culture, enhancing its relevance in the local context.
- Bilingual interviews allowed the participants to express themselves more comfortably, leading to more accurate and nuanced data.
- Controlling potential biases through a reflexivity log, verifying transcripts with authors and interviewees and involving multiple researchers in coding and theme development enhanced the reliability of the findings.
- The annual healthcare budget was accessed, but its segregation in terms of operating, administrative and other costs could not be analysed due to lack of data availability, which may affect findings’ credibility and validity.
- Conflicting opinions in interviews may complicate the analysis and interpretation of the findings.

## Introduction

 Forced migration is on a worrying rise worldwide. Global displacement hit a record high of 108.4 million people in 2022.^1^ Of these, 35.3 million people were refugees, the majority of whom are hosted in neighbouring low-income and middle-income countries (LMICs).^1^ While the United Nations General Assembly has committed to meet the basic health needs of refugees,^1^ the funding gap for UN-coordinated humanitarian appeals reached 65% in 2022.^2^

Nearly one million Rohingya people, a stateless Muslim ethnic minority group and one of the most persecuted communities in the world, have fled from Myanmar to Bangladesh and other neighbouring countries sporadically since the 1990s.^3^ The latest attack on the Rohingya by the Burmese military, described by the United Nations (UN) as ‘a textbook example of ethnic cleansing’, drove more than 700 000 Rohingya people to seek refuge in Bangladesh where they have been hosted with the support of the international community.^4^ Currently, there are an estimated 920 000 Rohingya people registered as refugees in Bangladesh, the majority of whom now live in 32 densely populated camps in Cox’s Bazar.^5^ More than 75% of these refugees are women and children, and up to 95% rely on humanitarian assistance. They have their own culture and dialect which is mainly blended, and partly similar to, both Bangladeshi people living in Cox’s Bazar and ethnic people living in Rakhine state of Myanmar. Following historical oppression by the Myanmar army-led government, the majority of the Rohingya people have been deprived of formal education, resulting in high illiteracy among all age group refugees.^6^ Critically, the refugee population makes up about one-third of the total population in Cox’s Bazar, highlighting the importance of supporting the host community. Recently, 24 000 Rohingya refugees have been relocated to Bhasan Char Island, a decision criticised by experts due to the isolated island’s vulnerability to environmental risks.^7^ Bangladesh has not ratified the 1951 Refugee Convention^8^ and has no specific laws guaranteeing refugees’ rights, leaving them vulnerable, with limited mobility and heavily reliant on aid.^9^

Bangladesh is a developing country, with a highly decentralised healthcare system. Public–private partnerships govern healthcare. International organisations, the Bangladesh government and other local partners provide healthcare.^10^ Healthcare in Bangladesh is largely dependent on out-of-pocket expenditure.^11^ Therefore, healthcare discrepancies are prominent all over the country.

Cox’s Bazar did not have adequate infrastructure to handle the needs of the local population.^12^ However, with the help of the international community, development partners and local organisations, infrastructure was developed after the mass Rohingya refugee influx occurred. Still, the equipment was scarce compared with demand. It should be noted that Bangladesh pays only 2.63% of its GDP on healthcare.^13^ Globally, host countries have adopted different care models to care for the refugees and migrant populations.^14 15^ These models include mainstream, specialised services, gateway services and limited access.^14 15^ While mainstream and mixed models of care model have been adopted in high-income settings, for example, Sweden, the USA, Germany and Australia, countries like Bangladesh have primarily offered limited access model, as characterised by high dependency on external aid and high workforce turnover whereas services are often provided in agreement with local policy-makers.^14 15^ However, Rohingya refugees in Bangladesh can also access, with support from local stakeholders, some mainstream services, for example, referral to tertiary hospitals for specialised treatments.^16^

The health needs of the Rohingya population are substantial and diverse due to their history of marginalisation, trauma, uncertainty, poor living conditions, limited water, sanitation and hygiene provisions and exposure to environmental risks (such as monsoons, cyclones and fires) in these camps.^16^ According to a recent review,^17^ the major health problems faced by Rohingya refugees include communicable diseases (eg, unexplained fever, diarrheal disease and acute respiratory disease) and chronic diseases such as hypertension. Due to the large population of women in the camps, sexual and reproductive health is also a crucial concern, with a particular need for antenatal care and support following gender-based violence.^18^ A surge in non-communicable diseases and mental illnesses is also being observed in camps.^19^ In recent years, elderly Rohingyas have reported difficulties in accessing routine medical care during the COVID-19 pandemic.^20^

The health response in Cox’s Bazar is led by a Health Sector Strategic Advisory Group with representatives from the Government of Bangladesh, UN bodies, national and international non-governmental organisations (NGOs) and is guided by a Joint Response Plan (JRP).^21^ The health response involves more than 100 partners from over 200 health facilities and targets 1.46 million people, including the host community.^16^ Initially, partners focused on establishing health facilities with baseline standards to ensure equity in service provision. Despite these standards, unequal distribution of health facilities remained, leading to a ‘rationalisation’ exercise in which subpar services were decommissioned or relocated.^16^ The overall emergency response was commendable, being capable of preventing potential health disasters, despite some limitations and challenges, for example, inadequate access to and utilisation of available services and a gradual decrease in funding for healthcare services.^22^

Implementation of health services is also impaired by social and environmental tension in Cox’s Bazar. The enormous scale of this crisis has put pressure on the livelihood of the local community,^8^ mirroring common conceptions that refugees drain the resources of their host nations^23^ and affect the economy negatively, for example, due to a decline in tourism, increase in the cost of commodities or rising poverty rates. However, this perspective overlooks the potential contribution of refugees to Bangladesh’s development by increasing workforce capacity, filling demographic gaps, increasing potential for bilateral trade and entrepreneurial activity, as seen in Rwanda’s refugee camps following the integration of refugees into economic activities.

The Rohingya refugee crisis is a highly complex predicament and requires partners to take a multidisciplinary, coordinated approach between international and local actors. Until there is an end to violence in Myanmar and safe, voluntary repatriation is feasible, high-quality healthcare must be accessible for the refugees. However, maintaining a balance in healthcare services for both refugees and local communities is challenging, especially with limited resources.

To effectively implement health programmes, initiatives have been taken to understand the burden and trends of disease,^17^ potential public health practices^24^ and primary healthcare utilisation^25^ among Rohingya refugees and adjacent host communities. Some studies have also highlighted the adverse impact of hosting Rohingya refugees on Bangladesh’s economy.^26^ However, healthcare financing mechanisms remain vastly underreported. To promote standardisation of the financing process and increase its effectiveness, it is essential to understand the divergent viewpoints of the stakeholders involved (UN bodies, NGOs and host government). This study aimed to increase the understanding of the healthcare stakeholders’ viewpoint on the challenges and potential solutions regarding healthcare financing for the Rohingya refugees in Cox’s Bazar.

## Methods

### Study design

We conducted a cross-sectional, mixed-methods study. For the qualitative component, we used an exploratory approach using eight semistructured interviews with healthcare stakeholders such as donors, implementers and coordination agencies/organisations.

For the quantitative component, administrative, financial and legal documents were reviewed and analysed to understand the funding gaps in healthcare, including documents available in the UN Office of the Coordination of Humanitarian Affairs (OCHA) and in the Office of the Refugee, Relief and Repatriation Commissioner (RRRC).^27^

### Settings and population

The study was conducted among eight healthcare stakeholders working in the humanitarian response for Rohingya refugees in Cox’s Bazar, Bangladesh. According to the latest JRP, there are currently 918 841 Rohingya refugees or Forcibly Displaced Myanmar Nationals in need of humanitarian aid. There are a total of 136 partners (10 UN agencies, 52 international NGOs and 74 Bangladeshi NGOs) responding through 178 humanitarian projects. The entire population is a target for the health sector response. After food security, health is the second-highest prioritised area, with 34 implementing partners contributing to the response through 27 projects.

### Participant selection

A maximum variation purposive sampling technique was used to identify participants with a diversity of perspectives. An initial list of potential participants was identified through a desk-based scoping exercise by a researcher with prior experience in the Rohingya context. Furthermore, healthcare service models adopted by Bangladesh, which is primarily a limited services model with conditional access to mainstream services, also provided insightful guidance in participant selection. Considering the modality of healthcare service delivery, participants were selected from government, non-government and UN organisations involved in the humanitarian response as donors, implementing partners and coordinating agencies (table 1).

**Table 1:** Sample characteristics

Organisation type	N
UN organisations	2
International Healthcare Programme Implementing Organisations	2
National Healthcare Programme Implementing Organisations	2
Host government healthcare administration officials	1
Host government refugee administration officials	1
	Total=8

Professional role in relation to refugee works	
Health programme manager	4
Programme executive	2
Policy and programmatic work	2
	Total=8

Academic background/training	
Clinical	1
Public health	6
Non-health	1
	Total=8

The sample size was guided by data saturation and information power. An assessment of information power revealed that a moderate sample size was required to address the study’s aims. Data saturation was assessed while no additional information was revealed from the study participants. Participants were informed about the study and invited to take part via email. Informed consent was collected electronically via email before interviews began (online supplemental file 1).

### Patient and public involvement

No patient or public was involved in any stage of this research. Research questions and outcome measures were developed by the research team through a literature review and from their prior expertise in this context. As the research was performed from a policy perspective, only the relevant key informants engaged in the health financing and implementation process were interviewed. Thus, no involvement of any patient or public was required in the entire process of the study design, data collection, and manuscript writing and dissemination plans of our research.

### Data collection and quality control

Data were collected through semi-structured interviews. Topic guides were prepared collaboratively by the study team, including two (IS and ST) who had previous experience conducting qualitative studies and two (MAR and JM) who had working experience in the Rohingya context (online supplemental file 2). Three female and two male researchers conducted the interviews. All interviews took place according to the schedule proposed by the study participants. The interviews were audio recorded and conducted online via Zoom. No repeat interviews were conducted. However, one participant did not consent to audio recording, so detailed hand notes were taken during that interview. In addition, one respondent refused to participate due to lack of interest. The suitability of the interview questions was confirmed through pilot interviews, which were not included in the final analysis. The interviews were conducted in both Bengali and English, based on participant preference. The mean duration of the interviews was 62 min. The interviews in English were transcribed using Otter electronic transcription platform (https://otter.ai/) and the transcriptions were checked, corrected and finalised by Bengali and English-speaking members of the study team. Bengali interviews were translated into English by bilingual members of the study team for coding and analysis. With the exception of one case, all interviews were conducted by at least two researchers. All researchers of the team received training on qualitative data collection and analysis prior to data collection and analysis.

To assess the gap in healthcare financing, document review involved only verified and trustworthy sources. The JRP available in the OCHA platform and documents shared with the RRRC office were included. The quantitative data for funding was taken from Financial Tracking Services by OCHA.^2^

### Reflexivity

The research team consisted of nine members. Among them, six (IHT, MAR, ST, JM, SSA and AFK) were Bangladeshi nationals with diverse occupational backgrounds, including physicians, academics, humanitarian workers, government employees and graduate students. Six (IS, PAV, IHT, MAR, PB and ST) researchers were international graduate students affiliated with Polygeia. Two (MAR and ST) were Bangladeshis with experience working in health and nutrition programmes at Rohingya refugee camps in Cox’s Bazar, Bangladesh. Two (IHT and AFK) team members were involved in policy implementation and coordination among different development sectors in Bangladesh.

To promote reflexivity, the researchers acknowledged their preconceptions before study initiation. They collaborated closely throughout data analysis to mitigate any impact of their prior experience and assumptions on the data analysis. No participant in this study was familiar with or previously communicated with any researchers.

### Potential bias control

In qualitative study, researchers’ preconceptions and involvements are the integral part of the information collection process, apparently posing a risk for potential bias, although reflexivity statements provide an insight into whether the degree of bias was controlled within acceptable limits. Still, several initiatives were taken to control the potential biases during the different stages of the study. As part of their role in the implementation and coordination of the host government policy, two authors (IHT and AFK) did not conduct interviews with local administrators in order to avoid being biased by the government’s predetermined viewpoints. Likewise, another author (JM) who initially contacted potential interviewees and was a part of healthcare implementation in Cox’s Bazar did not also participate in any interviews. Transcripts were sent to interviewees for feedback on whether they were correctly transcribed, so no information was expected to be distorted. Coding and theme development involved multiple authors, and disagreements were resolved through discussion.

#### Data analysis

The data were analysed using thematic analysis. In the initial data analysis stage, we used a code number for each participant to ensure the anonymity of the participant’s personal or organisational identity. After being familiarised with the data, each transcript was coded and cross-checked by two researchers. After initial coding, themes were identified based on emerging patterns in the codes by creating a coding tree using NVivo qualitative data analysis software. Final themes were discussed and agreed on by all researchers.

## Results

**Figure 1:**
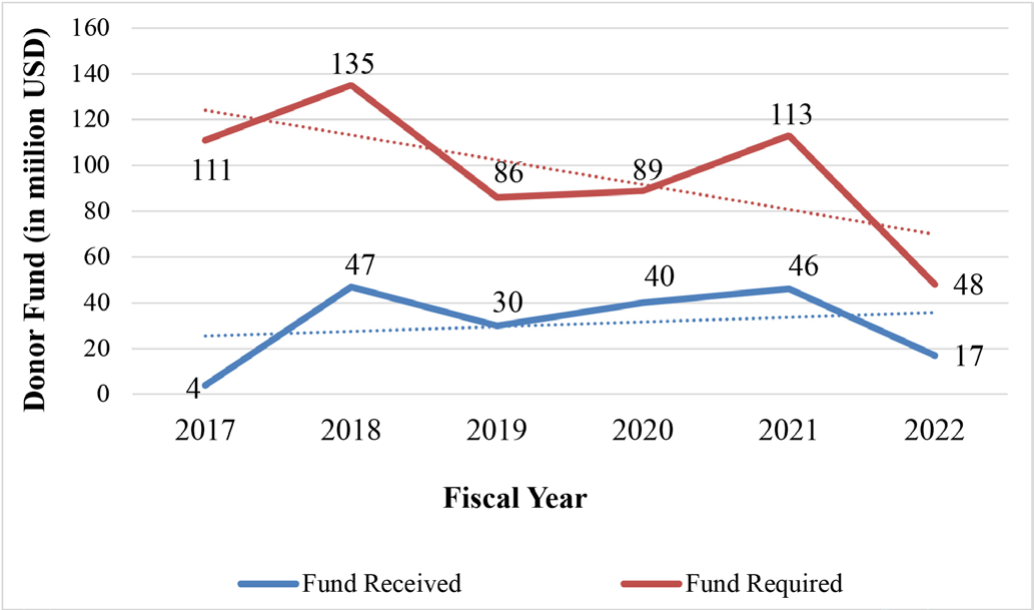
Fund required and fund received from 2017 to 2022. Source: Financial Tracking Service, United Nations Office for the Coordination of Humanitarian Affairs.^2^

Since the latest influx of Rohingya refugees to Bangladesh in 2017, there has been a significant gap between funds required and received by the implementing health agencies. The initial fund requirement was US$111 million, but donor aid totalled US$4 million. This gap has decreased with time but is yet to be closed. Between 2017 and 2018, funding requirements increased sharply from US$4 to US$47 million. Demand also increased accordingly. With reduced fund requirement and disbursement, this trend has been steady up to 2020, but rose again during 2020–2021. At the end of 2021 and 2022, this demand decreased again with universal immunisation and other pandemic interventions. However, a significant gap of about US$30 million still existed in 2022 (figure 1).

In our thematic analysis, we generated four themes and two subthemes which are discussed below.

### Theme 1: decreasing availability of global humanitarian assistance for healthcare delivery in the Rohingya community

Participants expressed their concern about dwindling financial support for the overall humanitarian response, specifically for healthcare. One disclosed, *“If you compare 2018 and 2019, financing is now reduced by 50%, or close to 60% according to the data of 2018… and donors are not releasing their funds for healthcare activities.”*

Many foresaw that financing would continue to decline in the future. Two participants from donor and implementing organisations discussed how crises in other countries, such as the war in Ukraine, are affecting the global economy and thereby impacting health financing in humanitarian contexts. Another respondent speculated that the graduation of Bangladesh from ‘least developed country’ status to middle-income status will severely affect its Rohingya response.

As a result of decreased funding, participants were concerned about their ability to deliver health services, with one reporting, “*Most partners couldn't even maintain their minimum funding requirements to continue critical health services.”*

Others revealed that many international organisations had narrowed their humanitarian work while some were abolished entirely due to limited resources. Despite these concerns, one lone participant was optimistic about donor organisations viewing healthcare as a priority.

Moreover, the respondents stated the funding comes from different sources, mostly from international donor agencies. The refugees also use the existing host government facilities through the referral process. However, there is no central and systematic pooling process of the allocated funds with a single administrative entity. Though the Inter Sector Coordination Group (ISCG) comprising the host government, donor agencies and implementing partners monitors the quality of service and provides strategic support to the whole system, it was reported by different interviewees that the finding is not pooled and allocated systematically. Thus, it creates programme overlapping, limits the scope of ensuring needs-based service assurance and irrational allocation of the resources to achieve the optimum healthcare outcome.

One respondent stated that the ISCG is the supreme authority to regulate all kinds of funding mechanisms and other directives for one fiscal year beginning from January to December. Therefore, every single organisation had to apply for permission from this group to use the budget and other administrative activities.

He also stated that each partner organisation shares their total plan to ISCG each year and the ISCG handles the respective sectors and controls organisational activities.

### Theme 2: conflicting short-term and long-term priorities are barriers to delivering durable health services

Respondents considered that differing priorities and uncertainty regarding the management of Rohingya response create difficulties in how healthcare is funded, prioritised and delivered in the long run.

When asked about sustainable solutions to healthcare financing, one participant summarised the issue, “*I usually find it difficult to answer the question on sustainability, because what are we trying to sustain here? This situation is not normal, right?”* This highlights how the refugee camps are intended as a temporary solution to accommodate the refugees, making long-term infrastructures elusive.

Most interviewees reported that short-term funding (over 1 or 2 years) was a barrier to implementing durable health programmes and suggested that the evolving nature of health needs requires long-term funding for better planning and organisation. One official from a donor organisation declared, “*We need to advocate for multi-year funding and development funding that strengthens the health system of the government in order to have a sustainable response.”* Employees from international and local organisations perceived existing temporary healthcare infrastructure as a persistent issue and suggested that increased financing could allow for more durable planning, including building more permanent health facilities.

While many reported that primary healthcare was widely available, some were concerned about insufficient aid for chronic or non-communicable disease care, secondary healthcare or specialist referrals within the camps. Respondents noted that demand for these services had increased over time as the acute emergency has transitioned into a protracted crisis.

In practice, very few donors had initiated multiyear funding. A participant from a donor agency revealed that the government does not permit the development of permanent health infrastructure in the refugee camps, stating, *“Maybe building a stone building with concrete is not a priority for the government, because that’s not what they want to sustain.”*

A long-lasting approach to managing the Rohingya community could improve healthcare financing and delivery while they remain in Cox’s Bazar. One worker from a donor organisation disclosed, “*We know everyone hopes that the refugees will be able to return to Myanmar soon. However, we need to ensure sustainable funding as long as they're here, while hoping that they'll be able to go back when the conditions allow for a safe, dignified, and voluntary return.”*

### Theme 3: achieving efficiency in healthcare funding is challenging in a complicated crisis

All participants noted the necessity for an efficient, needs-based approach to make best use of available funding. In fact, some participants (3/8) considered this to be the main challenge in healthcare financing, rather than a scarcity of funds. The absence of common processes as a cause of cost inefficiency was also highlighted.

#### Subtheme 3.1: collaborative priority setting enables a needs-based approach

Across the interviews, all participants from donor agencies perceived that coordination among the NGOs, donors, implementing agencies and the government was well established. Donors work with an international fundraising committee to raise funds and ensure their proper utilisation in healthcare. Needs-based funding is ensured through regular coordination meetings. Participants explained how healthcare is delivered in collaboration, with distinct roles being outlined for each partner.

In contrast, one government and one implementing NGO participant noted that donors and implementing NGOs do not engage the government and other local stakeholders in their project-planning phase or during implementation. Therefore, needs assessments were not always carried out in a manner that considered both host and refugee communities, leading to overlapping health programmes. Interviewees expressed that better coordination between these partners, enhanced monitoring and evaluation would allow clearer stewardship of programme funding and implementation.

Collaboration between local and international partners was viewed as particularly important. One respondent from a donor organisation divulged, *“There is comparatively less dependence on international actors to deliver health services since there is very good capacity at a national level. That being said, we still need international partners, because they bring in additional expertise.”* The respondent also recognised the importance of alignment with a common strategy. Another highlighted the importance of increasing local representation in the stewardship process, saying “*For implementing any project, they should engage the local representative, not only the ones in Dhaka (the capital city of Bangladesh). Also, the sponsor should oversee their project activities on a regular basis.”*

Participants mentioned that it was necessary to define priorities in order to improve efficiency of limited funds, with one employee from an international donor organisation suggesting, *“Identification and prioritization of needs is the starting point, followed by implementing activities that prove to be cost-efficient, evidence-based and effective in delivering those results”*.

#### Subtheme 3.2: implementing common processes can boost efficiency

Greater scrutiny of partners’ fund utilisation was seen as a critical part of improving efficiency of healthcare funding. Some spoke about high administrative costs, mentioning that some partners operated more efficiently than others. While there are guidelines in place outlining a ceiling of 20% for administrative costs, three participants (from government, international donor and implementing organisations) observed that many organisations exceed this level. However, two participants from implementing organisations mentioned that the ceiling is strictly maintained. One participant relayed that they were conducting a salary assessment to address discrepancies between partners. Another participant from an NGO recommended recruiting more employees from the local communities since their salaries are lower than overseas staff.

Many respondents, from donor, implementing and government organisations, affirmed having established monitoring activities. This included a financial tracking system that collected data on the amount and usage of funding and feedback from service users, shared information with the government and received government visits to ensure adherence to quality guidelines.

However, they also recognised potential to improve monitoring. A participant from a donor organisation reported that there is not enough scrutiny of the proportion of aid being spent on overhead costs and no systemic monitoring activities across partners.

Another suggested “*A proper, strong monitoring system should be established, both from the implementing partner and the donors.”* One participant expressed that there should be greater focus on scrutiny when selecting implementing partners in the first place.

An interviewee gave an example of an existing initiative to improve efficiency. They noted that there were more health centres in Cox’s Bazar than they were able to staff, leading to poor service. In response, the Strategic Advisory Group in the health sector was leading a rationalisation process that aimed to reduce the number of health centres from 150 to 80 to promote efficient utilisation of aid and enhance sustainability.

### Theme 4: lack of consensus on equity in healthcare services between the host and refugee communities

Maintaining equity between the host and refugee communities in Cox’s Bazar was seen as an important part of the humanitarian response. One respondent considered that investments in health system in the host community had strengthened social cohesion between the Bangladeshis and refugees, stating, *“Social cohesion is good for health.”*

While participants from donor and implementing agencies perceived that equity was ensured between these communities, the ones belonging to government agencies did not agree.

The Government of Bangladesh has stipulated that 25% of healthcare funding provided for Cox’s Bazar must be dedicated to supporting the host community. Participants from donor and implementing organisations reported that this provision is well maintained (and exceeded) across the health programmes in Cox’s Bazar. However, a government worker did not agree that 25% was always ensured and chalked it up to a loophole in funding allocation and the method of information being reported.

Participants from donor and implementing organisations reported that both communities have equal access to donor-funded healthcare services. They described how the host community can and do access services from health centres in refugee camps in Teknaf, “*Ask the refugees themselves. There’s no discrimination. We see people from those (host) communities coming to access our services as well in the camps.”* One respondent highlighted how there had been significant investment in improving government health services (the establishment of the first ICU in the district, a new specialist hospital and an outpatient complex), promoting better access to healthcare for both communities.

In contrast, one government official claimed that the host community has no access to the donor-funded care facilities, especially those who live far from the camps. They reported that resources were disproportionately allocated to the implementing NGOs in the refugee camps, reducing the number of staff at government healthcare facilities and challenging healthcare delivery for the host community. A participant from a donor organisation suggested that people from the host community feel entitled to access healthcare as they are Bangladeshi citizens, but donors prioritised providing services equally for both communities.

## Discussion

The objectives of the study were to understand stakeholders’ perceptions of healthcare financing challenges, filling a gap in existing evidence and exploring future options regarding healthcare financing for the Rohingya refugees in Bangladesh based on the recommended solutions. From the quantitative data and interviews, it is clear that funding for healthcare has decreased consistently since 2017, when the latest influx of Rohingya refugees into Bangladesh began. Participants from donor, implementing and government agencies felt that this decline, alongside coordination issues between partners, conflicting priorities and disagreements over ensuring equity, challenged the delivery of healthcare.

Deteriorating healthcare funding has been consistent over time, aggravated by global economic crises and mimicking an overall trend in humanitarian funding.^28^ The largest total gap in healthcare funding in Cox’s Bazar was seen in 2017, as huge capital expenditure was necessary to deal with emergency primary care needs and communicable diseases.^16^ The second sharp rise in the funding gap of 2020–2021 can be attributable to the COVID-19 pandemic, when access to isolation infrastructure, personal protective equipment, skilled manpower and education intervention in the Rohingya refugee camps were required.^29^ Participants identified how international emergencies, such as COVID-19 and the war in Ukraine, along with the graduation of Bangladesh to an LMIC, had impacted healthcare funding in Cox’s Bazar. Humanitarian needs are at a historically high level, with emergency appeals from the UN hitting US$51.5 billion in 2023.^30^ Participants reported that the shift in donor priorities away from the global south^31^ had led to many organisations limiting or halting their activities.

Over time, healthcare demands have shifted to a broader disease spectrum requiring long-term care. This corresponds with previous studies which have shown that the Rohingya population mostly suffers from NCDs like chronic obstructive pulmonary disease, chronic liver disease and community-acquired pneumonia.^32^ Along with decreasing overall funding, short-term funding for the health programmes was identified by most participants as a serious barrier to ensuring sustainable healthcare of refugees. Although the concept of sustainability was difficult to define in this context, participants expressed a clear conflict between short-term and long-term funding among the donor, implementing and government agencies. While most of the donor and implementing agencies shifted focus of healthcare from primary and communicable to non-communicable and specialist care, the government viewed long-term solutions as a threat to repatriation. The resolution of the 11th Meeting of the National Taskforce on Implementation of National Strategy on Myanmar Refugees and Undocumented Myanmar Nationals explicitly stated that permanent infrastructure would only be allowed in the Rohingya camps with prior permission of the national taskforce (online supplemental file 3). This reflects a concern that building permanent structures might express the host government’s willingness to accept the refugees as permanent residents and disincentivise refugees to repatriate since they have access to essential services and amenities.

Collaboration, coordination and engagement between stakeholders and weak stewardship mechanisms were identified as key challenges in the effective utilisation of healthcare funding. Participants recommended ensuring a common service delivery process based on a robust needs assessment to enable cost-effective health service delivery. Previous studies have also addressed the coordination gap and stakeholders’ conflicting interests as potential barriers to healthcare for the Rohingya refugees in Bangladesh.^9^ Existing research has identified that needs assessment data were not systematically included in the donors’ policy decision documents.^33^ One study^34^ revealed that in 2011, 100% of humanitarian aid in the Syrian crisis was spent unplanned; however, by 2019, plan-aligned fund utilisation had improved to 86%. These revelations are consistent with our study findings.

Contrasting equity perception of the refugee and host communities revealed that two different versions of equity discourse exist, characterised by counter-blaming and feelings of relative deprivation, further emphasising the lack of coordination between partners. Participants had differing views on whether the minimum percentage of aid-spending for the host community was being upheld. One study^7^ revealed that the host communities became resentful of Rohingya presence because of the termination of free medical checkups, stipend and counselling provided by different NGOs which were still available for the Rohingya population. This phenomenon can be explained through the lens of social identity theory^35^ and the theory of relative deprivation^36^ in the context of the refugee health system,^37^ which depicts the nature of group identity in the way of we versus them. In the Rohingya context, our findings reflected a perception of discrimination and mistrust among the host and refugee communities. Research exploring different forms of mistrust in the healthcare system in immigrant/refugee settings^38^ revealed perceived discrimination^39^ and poor health outcomes.^40^

Recommendations based on the findings of this study are as follows. First, collaboration and coordination mechanisms must be implemented to develop coherence among the actors and organisations. The United Nations High Commissioner for Refugees (UNHCR) and RRRC can be the focal entities generating collective effort in needs assessment and information-sharing among the stakeholders. This is expected to ensure the right mix of instruments, determine proper care delivery channels, customise different funds and engage donors strategically. Second, a standardised process is necessary as a direct outcome of that coherence. Third, introducing a crisis modifier in grant agreements is recommended to ensure flexibility of fund mobility between the strict borders of development and humanitarian aid. Flexibility of fund mobilisation is also being encouraged by donor agencies to build synergies between development and humanitarian aid. Finally, we advise a proper needs assessment with rational engagement of the host and refugee community representatives, implement the projects followed by those assessments, ensure transparency by intense monitoring and develop standard, specific guidelines through evaluation mechanisms.

### Strengths and limitations

Bangladeshi researchers in collaboration with international team members made it possible to fully understand the context of the study with cultural sensitivity and relevance. In addition to providing context-specific perspectives, multicultural and multisectoral team endeavours helped open the study up to a wider global perspective. All key informant interviews (KIIs) were bilingual, so accurate and nuanced data could be collected comfortably. By combining Bangladeshi native interviewers with foreign interviewers, the process became more objective, context-specific and relevant. We attempted to control potential biases by using a reflexivity log, verifying transcripts with authors and interviewees and involving multiple researchers in coding and theme development.

There are, however, a few limitations to the study. First, though the annual healthcare budget was collected and presented and triangulated through qualitative interviews, sector-wise expenditure and allocations, for example, operational, administrative and other costs were not possible to collect and present due to the lack of data availability. It might have impeded a more comprehensive understanding of the findings which may affect their credibility and validity. In addition, conflicting opinions provided by the KIIs in a few cases might have complicated interpretations. There were some points where the viewpoints of the KIIs from the host government and donor agencies differed. While multiple researchers were used during the coding and theme development in order to minimise possible misinterpretations, unusual misinterpretations could still occur.

## Conclusion

Healthcare financing in Rohingya refugee camps is characterised by multilayered complexities, driven by the potential challenges identified in this study. Healthcare policy must be systemic, evidence based, standardised and combinedly ventured by potential stakeholders to ensure sustainability. We recommend further studies focusing on each challenge with a closer lens, which could be enriched with the perspectives of macroeconomics and microeconomics, political science, and health policy.

## supplementary material

10.1136/bmjopen-2023-083021 online supplemental file 1

10.1136/bmjopen-2023-083021 online supplemental file 2

10.1136/bmjopen-2023-083021 online supplemental file 3

## Data Availability

Data are available on reasonable request.
