# Supplementary material for: Challenges of healthcare financing in the world’s largest refugee camp: a mixed-method study among healthcare stakeholders for Rohingya refugees in Bangladesh
Source: BMJ Open. 2025 Jan 23;15(1):e083021. doi: 10.1136/bmjopen-2023-083021 (PMC11784382; doi:10.1136/bmjopen-2023-083021)
Supplement: online supplemental file 1 [file bmjopen-15-1-s001.pdf]

# Informed Consent Form (in English)

**Title of the research:** Healthcare financing in the world's largest refugee camp: A mixed-method study among healthcare stakeholders for Rohingya refugees in Bangladesh.

Polygeia is a non-partisan student-run think tank engaging the brightest student minds studying in various universities and some researchers involved in research on global health issues and policies. It is based in the United Kingdom. This research activity is conducted by the Polygeia, Stockholm, Sweden branch engaging some volunteer students and researchers.

This information letter applies to study participants of this research project.

The main aim of this research project is to observe the types of health services provided, financial allocation and challenges related to financing as well as to take the opinion of the stakeholders and find out possible solutions to mitigate the challenges related to financing for the Rohingya refugee community.

**Name of principal investigator:** Syeda Saima Alam
**Designation:** Lecturer, Department of Food Technology and Nutrition Science, Noakhali Science and Technology University, Noakhali-3814, Bangladesh
**Mobile Phone Number:** +8801674828780, **Email:** saima.shoshi@gmail.com

There are two parts in the consent form:

1. Inform (to inform the participants about the research)
2. consent form

## 1st part: Inform

### Background

The international media has been publishing the news on Rohingya invasion and exploitation by the Myanmar military government since last century. In 2017, due to ethnic cleansing and annihilation, approximately 0.7 million Rohingya community took shelter in the Coxbazar district of Bangladesh. Since 1992, many Rohingya refugees have taken shelter in theCoxbazar district. In 2017, many Rohingya refugees also took shelter so the total 1.2 million Rohingya population is residing in Coxbazar district. For these huge amounts of Rohingya population settlement, international communities along with the Bangladesh government are working simultaneously. There have been many studies conducted to assess the burden of Rohingya refugees on the socio-economic, environmental, political pressure and other pressureinserted to the Bangladeshi host community. Moreover, many studies have conducted on refugee crisis in the low- and middle-income countries, the concern related to health and to mitigate the health

related necessary expenditures. However, it is quite impossible for the fragile health system and insufficient economy of a low- and middle-income country to manage health related expenditure for these refugee people. There are many existing studies related to the humanitarian crisis of the Rohingya refugees, however, studies related to finance are scarce. This research is aimed at understanding the health care financing, challenges and possible ways to mitigate the challenges related to health care financing for the Rohingya communities residing in Coxbazar district of Bangladesh as well as for the host communities Purpose:

1. To identify the total health care related required and spent financial cost and of the Rohingya refugee influx from 2017.

2. To explore the highest prioritized area for healthcare financing. To identify the areas that need prioritization and need more allocation.

3.To understand the international donor agencies related to healthcare financing and to identify their prioritized areas for funding.

4. To compare the difference between the financial allocation between host and the Rohingya community.

5.To explore the challenges in financing for the Rohingya community.

6. To assess the sustainability of this financing and the risk related to the financing.

7. Recommendation of the stakeholders to mitigate the healthcare financing challenges

**Data collection procedure**

This research activity will take place by taking consideration of the secondary information from the relevant offices and from the interviews conducted with the stakeholders. We will collect information about the Rohingya community from the existing scientific papers, reports and financial services and other information from the Rohingya relief return commissioner office (RRRC). The other information will be collected using Key Informant Interviews.

**Participants selection process**

As you are a health care service related stakeholder so your experience will be helpful to fulfill our research purpose. So we selected you purposively.

**Willingly participation**

You have the right to decide whether or not to participate in this research. You have the right

to withdraw yourself at any time during the interview of this research study. We will provide the guidelines to the participants prior to the interview, so that the participants get to know about the purpose of this research project and can think or take the opinions from their relevant authority to make a decision to participate in this research. During the interview if the participants do not want to answer any question, they can avoid it. In this case the interviewer will proceed to the next question. The interview will take place at the convenient place suggested by the participants. Only the researcher and the participant will be present during the interview or if the participants allow anyone she or he can be present. All the given and registered information is personal and confidential. It will be ensured that the information will only be accessed by the researchers.

**Risk and Disadvantages**

There is no risk in participation in this research. We will conduct the interview according to your convenience and all your given information will be used only for research purposes. None of the given information will be revealed individually, all your identity and job related information will be kept highly confidential.

**Possible Advantages**

There is no individual benefit in taking part in this research project. But your given information will be used to create advanced policy regarding health financing. We will conduct the interview according to your preferred schedule.

**Confidentiality**

The information about the participants will not be shared with anyone outside the research group. Personal information about the participants will be kept confidential by using coding and will not be used in any purpose other than the research. The information will not be shared with anyone outside the research group.

**Disclosure of research findings**

The research findings will be published as a research paper.

**Contact**

If the participants have any queries regarding this research project, they can ask anytime to the researchers involved in this project. Information can be retrieved even after the research project started or the information has been registered. The participants can contact in the below

mentioned contact numbers.

Name:

Designation:

Phone number:

Email:

**2nd part: consent form**

I read the previous information to participate in this research and I have clearly understood about this research project. I got an opportunity to ask questions and I got a satisfactory answer. I, as a participant, agreed to participate in this research project willingly.

Name:

Signature or fingerprint

Date:

Researchers statement:

I read this consent form to the participants when required and I tried my level best to make the participant understand about this research project.

I can firmly state that the participants were not forced to participate in this research project and participants provided their consent independently and willingly.

Name:

Designation:

Phone number:

Email:

# Informed consent form in Bangla

# সম্মতি পত্র

গবেষণার শিরোনামঃ Healthcare financing in the world's largest refugee camp: A mixed-method study among healthcare stakeholders for Rohingya refugees in Bangladesh

এই গবেষণাটি বিশ্বব্যাপী গ্লোবাল হেলথ সম্পর্কিত গবেষণার সাথে সংশ্লিষ্ট বিভিন্ন বিশ্ববিদ্যালয়ের শিক্ষার্থী ও গবেষকদের সমন্বয়ে গঠিত যুক্তরাজ্যভিত্তিক আন্তর্জাতিক থিংক ট্যাংক Polygeia, Stockholm, Sweden শাখা কর্তৃক পরিচালিত একটি স্বেচ্ছাসেবক (volunteer) শিক্ষার্থী ও গবেষকদের কর্তৃক পরিচালিত একটি গবেষণা।

গবেষণায় অংশগ্রহণকারীদের এই অবগতি পত্র প্রযোজ্য। এই গবেষণার বিষয়বস্তু হলো রোহিঙ্গা শরনার্থীদের জন্যে স্বাস্থ্যসেবার ধরণ, বরাদ্দকৃত আর্থিক সংস্থান, এবং আর্থিক সংস্থানের ক্ষেত্রে বাধাসমূহ ও তাঁর সমাধান সম্পর্কিত সংশ্লিষ্ট স্টেকহোল্ডারদের মতামত পর্যবেক্ষণ করা।

প্রধান অনুসন্ধানকারীর নামঃ

পদবীঃ

প্রতিষ্ঠানের নাম ও ঠিকানাঃ

এই সম্মতি পত্রে দুটি অংশ বর্তমানঃ

১। অবহিতকরন (অংশগ্রহণকারীদের সাথে গবেষণা সম্পর্কে তথ্য প্রদান)

২। সম্মতি পত্র

## প্রথম অংশঃ অবহিতকরন

**ভূমিকাঃ**

গত শতাব্দী থেকেই মায়ানমার সামরিক সরকার কর্তৃক রোহিঙ্গা জনগোষ্ঠীর উপর শোষণ ও নির্যাতনের খবর আন্তর্জাতিক সংবাদমাধ্যমের দ্বারা বিশ্বদরবারে প্রকাশিত হয়ে আসছে। মায়ানমারে জাতিগত নিধন ও আগ্রাসনের ফলে ২০১৭ সালে প্রায় ৭ লক্ষ রোহিঙ্গা শরণার্থী বাংলাদেশে আশ্রয় গ্রহন করে। বাংলাদেশের কক্সবাজার জেলায় ১৯৯২ সাল থেকেই অনেক রোহিঙ্গা শরনার্থী আশ্রয় গ্রহন করে। ফলে ২০১৭ সালে পুনরায় ব্যাপক শরনার্থী সমাগমের ফলে প্রায় ১.২ মিলিয়ন রোহিঙ্গা শরনার্থী কক্সবাজারে আশ্রয় গ্রহন করে। এই বিশাল শরনার্থীর বসবাসের লক্ষ্যে বাংলাদেশ সরকারের পাশাপাশি আন্তর্জাতিক সম্প্রদায় ও কাজ করছে।এই বিশাল রোহিঙ্গা জনগোষ্ঠীর সৃষ্ট আর্থ-সামাজিক, পরিবেশগত, রাজনৈতিক চাপসহ বাংলাদেশে আশ্রয়দানকারী জনগোষ্ঠার উপর নানা ধরণের প্রভাব নিয়ে বিভিন্ন গবেষণা পরিচালিত হয়েছে। বিশ্বব্যাপী নানা গবেষণায় দেখা গিয়েছে যে নিম্ন ও মধ্যম আয়ের দেশগুলোতে শরনার্থী সমস্যায় আশ্রিত রোহিঙ্গাদের একটি অন্যতম ভাবনার বিষয় হয়ে থাকে তাদের স্বাস্থ্য এবং এর পেছনে প্রয়োজনীয় ব্যায় মেটানো যা নিম্ন ও মধ্যম আয়ের দেশগুলোর ভঙ্গুর স্বাস্থ্য ব্যাবস্থা ও অপ্রতুল অর্থনীতি দিয়ে মেটানো সম্ভব হয়না। মানবিক বিপর্যয়ে বিপন্ন রোহিঙ্গা

শরনার্থীদের স্বাস্থ্য নিয়ে নানা ধরণের গবেষণা থাকলেও এ খাতে অর্থায়ন নিয়ে তেমন কোন গবেষণা চোখে পড়েনা। তাই বর্তমান গবেষণাটি বাংলাদেশের কক্সবাজার জেলায় অবস্থানরত রোহিঙ্গা জনগোষ্ঠী ও তৎসংলগ্ন বাংলাদেশী জনগোষ্ঠীর স্বাস্থ্যখাতে অর্থায়নের প্রক্রিয়া, চ্যালেঞ্জসমূহ, এবং এর থেকে উত্তণের সম্ভাব্য প্রক্রিয়াসমূহ চিহ্নিত করার নিমিত্ত পরিচালিত হবে।

**উদ্দেশ্যঃ**

১) ২০১৭ সালের সর্বেশেষ ব্যাপক আকারে রোহিঙ্গা শরনার্থী আগমনের সময় হতে বর্তমান সময় পর্যন্ত বছর অনুযায়ী স্বাস্থ্যখাতে প্রয়োজনীয় ও ব্যায়িত অর্থের পরিমাণ।

২) সর্বোচ্চ প্রাধান্যপ্রাপ্ত বরাদ্দের খাত সম্পর্কে ধারণা গ্রহন। কোন কোন খাতে আরও বরাদ্দ এবং প্রাধান্য দেওয়া প্রয়োজন তা সম্পর্কে ধারণা গ্রহন।

৩) স্বাস্থ্যসেবায় অর্থায়কারী বিভিন্ন দাতা সংস্থা এবং অর্থায়নের ক্ষেত্রে তাঁদের প্রাধান্য সম্পর্কে ধারণা গ্রহন।

৪) রোহিঙ্গা শরনার্থী ও স্থানীয় বাঙ্গালী জনগোষ্ঠীর প্রাপ্য স্বাস্থ্য অর্থায়ন ও সেবা প্রাপ্তির তুলনামূল চিত্রায়ণ।

৫) রোহিঙ্গা শরনার্থীদের স্বাস্থ্যখাতে অর্থায়নের চ্যালেনজসমূহ।

৬) এ অর্থ্যায়নের টেকসইকরণ ও এক্ষেত্রে সম্ভাব্য ঝুকিসমূহ কী কী?

৭) স্বাস্থ্য অর্থায়নের সাথে জড়িত স্টেকহোল্ডারদের দৃষ্টিতে এ চ্যালেনজসমূহ মোকাবেলায় টেকসই সুপারিশমালা কী কী হতে পারে?

**গবেষণার তথ্য সংগ্রহের ধরনঃ**

এই গবেষণাটিতে দাপ্তরিক প্রাপ্ত তথ্যাদি ও স্টেকহোলডারদের সাক্ষাৎকার গ্রহণের মাধ্যমে পরিচালিত হবে। রোহিঙ্গা কম্যুনিটির স্বাস্থ্য সেবায় অর্থায়ন সংক্রান্ত প্রকাশিত প্রতিবেদন, বৈজ্ঞানিক গবেষণাপত্র ও শরনার্থী ত্রাণ ও প্রত্যাবাসন কমিশনারের কার্যালয়, কক্সবাজার হতে প্রাপ্ত আর্থিক, সেবা ও অন্যান্য সংশ্লিষ্ট তথ্যের পাশাপাশি এই গবেষণায় সাক্ষাৎকারের মাধ্যমে তথ্য সংগ্রহ করা হবে।

**অংশগ্রহনকারীদের বাছাই প্রক্রিয়াঃ**

আপনি যেহেতু  স্বাস্থ্যসেবার সাথে সম্পর্কিত একজন স্টেকহোলডার সেহেতু আপনার অভিজ্ঞতা আমাদের গবেষণার উদ্দেশ্য পূরনে সহায়ক হবে। সুতরাং, আপনাকে উদ্দেশ্যমূলক ভাবে নির্বাচন করা হয়েছে।

**স্বেচ্ছায় অংশগ্রহণ**

এই গবেষনায় অংশগ্রহন করার বা না করার সিদ্ধান্ত নেয়ার সম্পূর্ন অধিকার আপনার রয়েছে। সাক্ষাৎকারের যে কোন সময়ে যেকোন আপনি সাক্ষাৎকার প্রদান থেকে নিজেকে প্রত্যাহার করার অধিকার রাখেন। অংশগ্রহনকারীরা যেন এই

গবেষণার উদ্দেশ্য সম্পর্কে জানতে পারে, এটি নিয়ে চিন্তা করতে পারেন ও প্রয়োজনে কর্তৃপক্ষের মতামত নিতে পারেন সে জন্য এই তথ্যপত্রটি তাদেরকে অগ্রিম দেওয়া হবে যাতে তারা তাদের সিদ্ধান্ত জানাতে পারেন।

সাক্ষাৎকার চলাকালীন সময়ে কোন অংশগ্রহনকারী যদি কোন প্রশ্নের উত্তর দিতে না চান, তবে তিনি তা এড়িয়ে যেতে পারেন। এক্ষেত্রে সাক্ষাৎকার গ্রহনকারী পরবর্তী প্রশ্নে এগিয়ে যাবেন। সাক্ষাৎকারটি অংশগ্রহনকারীর সুবিধাজনক স্থানে অনুষ্ঠিত হবে এবং সাক্ষাৎকার গ্রহনকারী ও অংশগ্রহনকারী ব্যাতিত কেউই সেখানে থাকবেন না, যদি না অংশগ্রহনকারী অন্য কাউকে উপস্থিত থাকতে বলেন।

প্রদানকৃত ও নথিভুক্ত তথ্য ব্যক্তিগত ও গোপনীয়। ফলে কার্যদর্শী ও গবেষক ব্যতীত অন্য কেউ যেন এই তথ্য ব্যবহার করতে না পারে তা নিশ্চিত করা হবে।

**ঝুঁকি ও অসুবিধাঃ**

এই গবেষণায় অংশগ্রহনে আপনার কোন ঝুকি নেই। আমরা আপনার সুবিধামত সময়ে আপনার সাক্ষাতকার নিবো এবং আপনার দেয়া তথ্য কেবলমাত্র গবেষণার কাজে ব্যবহৃত হবে। ব্যক্তিগত পর্যায় থেকে প্রাপ্ত তথ্য কোনভাবেই এককভাবে প্রকাশ করা হবে না এবং আপনার পরিচয় এবং চাকুরি সংক্রান্ত তথ্য সম্পূর্ণ গোপন রাখা হবে।

**সম্ভাব্য সুবিধাঃ**

এই গবেষনায় অংশগ্রহণে আপনার ব্যক্তিগত কোন সুবিধা হবে না। তবে আপনার দেওয়া তথ্য  ভবিষ্যতে নতুন ও উন্নত নীতিমালা তৈরি করতে সহায়তা করবে। আমরা আপনার সুবিধাজনক সময়ে আমরা আপনার সাক্ষাৎকার নিবো।

**গোপনীয়তাঃ**

অংশগ্রহনকারীদের সম্পর্কে কোন তথ্য গবেষক দলের বাইরে অন্য কাউকে প্রদান করা হবে না। কোডিং এর মাধ্যমে আমাদের এই গবেষণা প্রকল্প থেকে সংগ্রহ করা ব্যক্তিগত তথ্য গোপন রাখা হবে। ফলে সংগৃহীত তথ্য গবেষক ব্যাতীত অন্য কেউ দেখতে সক্ষম হবে না।

**গবেষণার ফলাফল প্রকাশঃ**

গবেষনা থেকে প্রাপ্ত ফলাফল গবেষণাপত্র আকারে প্রকাশ করা হবে।

**যোগাযোগঃ**

যদি সংশ্লিষ্ট বিষয়ে অংশগ্রহনকারীদের কোন প্রশ্ন থাকে, তবে যেকোন সময় তাঁরা সেটি গবেষকদের জানাতে পারবেন। এমনকি গবেষনা শুরু হবার পরে বা তথ্য নথিভুক্ত হবার পরেও তা প্রযোজ্য। অংশগ্রহনকারীগন নিম্নলিখিত মাধ্যমে যোগাযোগ করতে পারেন।

**নামঃ** Syeda Saima Alam
**পদবীঃ** Lecturer, Department of Food Technology and Nutrition Science, Noakhali Science and Technology University, Noakhali-3814, Bangladesh

**ফোন নম্বরঃ** +8801674828780

**ই-মেইলঃ** saima.shoshi@gmail.com

# দ্বিতীয় অংশঃ সম্মতি পত্র

**সম্মতি পত্র**

এই গবেষনায় অংশগ্রহনের জন্য আমি পূর্ববর্তী তথ্য পড়েছি এবং আমি সম্পূর্ন বিষয় বুঝতে সক্ষম হয়েছি। আমি এই বিষয়ে প্রশ্ন করার সুযোগ পেয়েছি এবং আমাকে সন্তোষজনক উত্তর দেওয়া হয়েছে। আমি এই গবেষনায় অংশগ্রহনকারী হিসেবে স্বেচ্ছায় অংশগ্রহন করতে সম্মত।

**নামঃ**

**পদবীঃ**

**ফোন নম্বরঃ**

**ই-মেইলঃ**

**স্বাক্ষর বা ছাপঃ**

**তারিখঃ**

**গবেষকের বিবৃতিঃ**

প্রযোজ্য ক্ষেত্রে আমি সম্মতি পত্রটি সঠিকভাবে পড়ে শুনিয়েছি এবং অংশগ্রহনকারী যেন গবেষণার উদ্দেশ্য সম্পর্কে বুঝতে পারে সেই বিষয়ে সর্বোচ্চ সচেষ্ট থেকেছি।

আমি নিশ্চিত করে বলছি যে, কোন অংশগ্রহনকারীকে সম্মতি প্রদানের ক্ষেত্রে জোর করা হয়নি এবং সম্মতিটি স্বতন্ত্রভাবে ও স্বেচ্ছায় দেওয়া হয়েছে।

**নামঃ** Syeda Saima Alam

**পদবীঃ** Lecturer, Department of Food Technology and Nutrition Science, Noakhali Science and Technology University, Noakhali-3814, Bangladesh

**ফোন নম্বরঃ** +8801674828780

**ই-মেইলঃ** saima.shoshi@gmail.com
