# Supplementary material for: Challenges of healthcare financing in the world’s largest refugee camp: a mixed-method study among healthcare stakeholders for Rohingya refugees in Bangladesh
Source: BMJ Open. 2025 Jan 23;15(1):e083021. doi: 10.1136/bmjopen-2023-083021 (PMC11784382; doi:10.1136/bmjopen-2023-083021)
Supplement: online supplemental file 2 [file bmjopen-15-1-s002.pdf]

Topic guide/ Interview guidelines

Introduction:

Thank you for agreeing to speak with me today and for giving us your time.

I am "X" from Polygeia Stockholm Branch, a global health think tank led by students.

We are interested in understanding your views on healthcare financing for the Rohingya community, as this is currently a research gap.

We are interested in the perceptions of the implementing stakeholders about challenges regarding healthcare financing in this humanitarian context.

Before we begin, can I check that you have received and signed an informed consent form? (Y/N)

If NO:

- *This interview forms part of a study on the challenges regarding healthcare financing for the Rohingya refugee community within Bangladesh. We will analyse and write up the findings and present them at the Polygeia conference in October.*
- *The interview will take no more than 1 hour. We will ask you questions regarding your perspectives on gaps and challenges with health financing in the Rohingya setting.*

- *Identifiable information will not be shared. Your responses to the questions will be analyzed by the research team, stored securely, and not shared more widely.*
- *I understand that my participation in the study is voluntary and that I am free to withdraw at any time, without giving any reason.*

Do you mind if I record this interview? The recording will not be shared outside of the research team. Do you have any questions?

Topic 1: Introduction and overview of health (financing) system

1. **What role does your organisation play in providing healthcare for Rohingya refugees and/or the host community?**
    a. What is your individual role?
    b. What is your specific area of focus? (prompt: area of healthcare, population)
2. **Can you describe how you/ your organisation fits into the broader healthcare system, in your view?**

   OR, How is your organization supporting the healthcare system of Bangladesh?
    a. Prompts: financing, services, service delivery
    b. How do the different organisations work together to provide/fund healthcare?

Topic 2: Rohingya healthcare **compared to host community**

3. **To what extent do you think the Rohingya community utilizes and gets access to healthcare?**

4. **To what extent do you think the host community utilizes and gets access to the healthcare system?**
5. **Is there any difference in accessing healthcare between refugee and host community?**

6. Tell us about the health financing budget to meet the needs of the both Rohingya and host communities (in terms of healthcare utilization)?

   Antenatal, reproductive health, Chronic and NCDs?

Topic 3: Challenges

7. **In your opinion, can you tell us about the gaps in the funding of healthcare for Rohingya refugees?**

   Separate funding from the govenment only devoted for the Rohingyas?

   a. Prompt: are there particular diseases which are underfunded?
   b. New question: Tell us about how the funding is utilised - **are there any ways the use of the funds could be improved? If so, how?**
8. **Please describe challenges in financing healthcare for the Rohingya and host communities.**

   Prompts: lack of international funding, rising threat of specific diseases, environmental challenges, socio-economic pressure on host community, sexual and reproductive health, crime.

Topic 4: Recommendations

9. **What are your recommendations addressing the gaps in funding healthcare for refugees?** *This could be a sub-question for 6, or a prompt for question 9.*

10. **In your view, what are the sustainable solutions to the health crisis of the Rohingya community?**
    a. Prompts: capacity building, integration, voluntary repatriation, official refugee status

Additional topics covered during interview:

Funding transparency, accountability, how to ensure equity in health financing

Bhasanchar issue

Social and health burden to host community and country. Your perception

1. There is a disparity in seeking health care among the males and females. Especially, sexual and reproductive health is often not be considered as a problem in the Rohingya community. How do you perceive this as a problem? If there any! Is it creating any additional burden in the health care system?

2. According to your perception, what are the diseases that creating burden in the health system?

3.To your knowledge, do you have any idea about the host community displacement taken place due to the Rohingya settlement (as the Rohingya's creating social problems)?

11. Is there anything else related to funding healthcare for Rohingya that you would like to share?

Closing Comments: Do you have any further comments or questions? Would you be available if I contact you for further clarification? How would you like to be contacted – via email or Zoom? Thank you very much for sharing your opinions and experiences with me today.
