# Supplementary material for: Challenges of healthcare financing in the world’s largest refugee camp: a mixed-method study among healthcare stakeholders for Rohingya refugees in Bangladesh
Source: BMJ Open. 2025 Jan 23;15(1):e083021. doi: 10.1136/bmjopen-2023-083021 (PMC11784382; doi:10.1136/bmjopen-2023-083021)
Supplement: online supplemental file 3 [file bmjopen-15-1-s003.pdf]

Minutes of the Twenty-First Meeting of the National Taskforce on implementation of the National Strategy Paper on Myanmar Refugees and Undocumented Myanmar Nationals

The twenty-first meeting of the National Task Force (NTF) on Implementation of the National Strategy on Myanmar Refugees and Undocumented Myanmar Nationals (UMNs) was held on 14 June 2018 at the Ministry of Foreign Affairs. Mr. Md. Shahidul Haque, Foreign Secretary presided over the meeting as the Chair of NTF. The list of participants at the meeting is furnished at **Annex-A.**

2. The meeting was convened primarily to discuss the impending projects in Cox's Bazar with the funding from World Bank and Asian Development Bank. In addition to that the meeting also discussed current situation with humanitarian response, progress in repatriation and overall law and order situation in Cox's Bazar. The agenda of the meeting is enclosed at **Annex B**.

3. UN Resident Coordinator updated the meeting that only 20% of the funding has so far been secured for implementing Joint Response plan (JRP) that outlines humanitarian response for the forcibly displaced Rohingyas from March to December 2018. However efforts are ongoing to mobilize rest of the necessary funding, she added. The representative of the UNHCR was then requested to brief the meeting about the recently concluded MOU between UNHCR and Myanmar. UNHCR representative informed that the MOU has addressed the issue of right to safe and dignified return, choice of place for settlement, right to citizenship, legal, material, physical safety of returnees, freedom of movement and issue of access of UN and other humanitarian actors in Rakhine. He also stressed on the implementation of the MOU on the ground for its effectiveness. The IOM representative then briefed the NTF members on the sector-wise implementation status of the humanitarian assistance programme and the current challenges including those posed by heavy rains. They also informed the meeting about the progress in relocation of vulnerable Rohingyas. The UN particularly stressed on the need to strengthening the shelters, and access to the camps.

4. The Foreign Secretary then invited the ERD & World Bank representatives to brief the meeting about the World Bank's plan of action. It was known that –

- At the request from Hon'ble Finance Minister, World Bank decided to provide financial support for the forcibly displaced Myanmar nationals (FDMNs) and the host communities in Cox's Bazar under its IDA-18 Regional Sub-window for Refugees and Host Communities. Although IDA's established basis is 50% credit and 50% grant, considering the scale of the Rohingya influx and its potential impact on Bangladesh's own development, the World Bank decided to convert the entire assistance into grant.
- Under this arrangement, 5/6th of the project cost would be provided by World Bank while 1/6th would be funded from Bangladesh's national IDA on terms applicable for Bangladesh for which WB is trying to secure additional resources

from other donors to buy down the portion of project costs and make it fully financed on Grant terms. The Bank would assist the GoB in providing support for the host communities through existing operations and potential new funding mechanisms.

- The size of WB's grant would be USD 400 million in total. In the first phase, the WB support will include the following interventions: a. USD 50 million Health Project to be facilitated through the MoH&FW; b. USD 130 million Education Project to be facilitated through the MoPME for expansion of basic numeracy and literacy programs, as well as life skills training to out of school children; c. Multi-sectoral Rohingya Support Project to be facilitated through the LGD, combining WASH (USD 125 million), community resilience (USD 40 million) and institutional strengthening (USD 5 million).
- World Bank has submitted its proposal on the Health Sector Support Project to the ERD, which is worth of USD 50 million (41.67 million grants and 8.33 million credits). The negotiations between GoB and World for the funding will be held today (on 14 June 2018) and accordingly the proposal would be placed before eth World Bank Governing Board on 18 June 2018. WB would like to launch the project on 27 June 2018.

5. The meeting discussed about the coordination mechanism envisaged for the implementation of the WB projects. Although the WB representative said that the WB projects would be built on existing humanitarian response led by the UN and they would duly coordinate with the implementing Ministries /agencies, it was not clear how the interface would look like between the WB and the existing coordinating entities, such as, Strategic Executive Group (SEG), Inter-Sectoral Coordination Group (ISCG) and the National Task Force (NTF). The representative of Public Security Division also opined that there should not be any parallel mechanism; rather they could develop an *Operation Plan* within the existing coordinating mechanism to ensure that the implementation is done smoothly and with minimum cost.

6. After the discussion on WB Plan of Action, the NTF provided following views -

i) The WB projects should be implemented in an integrated manner under the existing coordination mechanism to ensure effective service delivery and to prevent duplicity of response and misgivings/ confusions among different UN and GoB stakeholders.

ii) The Rohingyas are forcibly displaced nationals of Myanmar and has not been declared/identified as "refugees" by the Government of Bangladesh. The GoB is currently focusing on safe and sustainable return of the Rohingyas to Myanmar and accordingly working with international community towards creating conducive environment in Rakhine. Therefore, WB must not take any long-term project for the Rohingyas.

iii) The full project document should be submitted to the NTF. The NTF and concerned Government entities should be consulted for education related projects and other projects funded by the World Bank.

7. After the discussion on the World Bank Projects, the Chair invited the representatives of ERD and ADB to brief the meeting about the ADB funded projects. It was known that:

- At the request of Finance Ministry, the ADB sent a fact finding mission to Bangladesh from 03-07 June 2018 to assess needs for interventions in Cox's Bazar through ADB funding. The President of ADB also paid a visit to Bangladesh in February 2018 and met with HPM. Unlike WB, ADB does not have any 'refugee' programme. They have therefore decided to provide the assistance in the form of its 'Emergency Assistance Project'.
- ADB is considering providing funding up to $200 million, with $100 million for the first phase. The Government will provide $20 million equivalent to cover (i) taxes and duties, and (ii) land acquisition if any and project management /counterpart staff.
- ADB support will be focused, selective, and well-targeted in the areas of (i) road access to and within the camps; (ii) water and sanitation; (iii) energy supply; and (iv) disaster risk mitigation. The projects will be implemented in Ukhiya and Teknaf.
- NTF would be the top coordinating body. The coordination at the local level would be done through the Office of the RRRC under the Ministry of Disaster Management and Relief (from GoB side) and the ISCG, ox's Bazar under the leadership of Strategic Executive Group co-led by UNRC, UNHCR and IOM.

8. During discussion the representative of PSD opined that the project locations may be extended to conceive possible cooperation in Bhashan Char. Referring to the severe crisis of drinking water in Cox's Bazar, the NTF Chair advised that the ADB should develop a reservoir in Cox's Bazar to meet the demands of Rohingyas and the host communities. The meeting also recognized that the coordination mechanism proposed by the ERD-ADB team looked sound (enclosed as Annex C). The Chair suggested that the NTF should not be equated with Ministry of Foreign Affairs; it is an inter-ministerial body and MoFA only provides secretarial service to the body. The Meeting decided to convey 'no objection' to the ADB's 'Aide Memoire'.

9. Among other issues, the meeting discussed about a recent incident of irregular movement by sea of a boat which ended up in Buthidaung, Myanmar. Mostly the Rohingyas were travelling through the boat; however some Bangladeshis are also believed to be there. The Chair advised the Ministry of Home Affairs, local administration and the Coast Guard to increase vigilance to prevent any trafficking attempt by the organized groups. The Chair also informed the meeting that the UN Secretary General and the World Bank President would visit Cox's Bazar on 1 July 2018 for which the cooperation from local administration would be extremely important.

10. In course of discussions, the meeting took the following decisions:

a) The aide memoire of ADB regarding its projects in Cox's Bazar along with the proposed coordination mechanism has been approved by the NTF.
b) The Plan of Action of the World Bank was approved with the understanding that it would streamline its coordination mechanism with the existing entities - NTF, SEG and ISCG (as enclosed in Annex C).
c) The World Bank and the ADB would not take any long-term project especially related to formal education and employment generating activities for the Rohingyas while they are in Bangladesh.
d) The World Bank would share full project documents regarding its intervention in Cox's Bazar with the NTF.
e) The World Bank would implement the education and other projects upon concurrence from concerned stakeholders including the NTF.
f) The Ministry of Foreign Affairs, Cox's Bazar district administration and the law enforcing agencies would increase vigilance to prevent human trafficking from Cox's Bazar.

11. With no other issues to be discussed, the Chair declared the meeting closed.

(Md. Shahidul Haque)

Foreign Secretary and Chair,
National Task Force on Implementation of National Strategy on Myanmar Refugees and Undocumented Myanmar Nationals

Minutes of the Twenty-Second Meeting of the National Taskforce on implementation of the National Strategy Paper on Myanmar Refugees and Undocumented Myanmar Nationals

The twenty-second meeting of the National Task Force (NTF) on Implementation of the National strategy on Myanmar Refugees and Undocumented Myanmar Nationals (UMNs) was held on 06 September 2018 at the Ministry of Foreign Affairs. Hon'ble Foreign Minister graced the meeting and briefed the meeting on his recent visit to Myanmar. He outlined some policy key directions regarding the Rohingya issue. Later, Mr. Md. Shahidul Haque, Foreign Secretary and the Chair of the NTF presided over the meeting. The list of participants at the meeting is furnished at Annex-A.

02. The meeting discussed the diplomatic efforts and progress made in repatriation, current situation with humanitarian response, the impending emergency humanitarian assistance projects of the World Bank and the ADB, and overall law and order situation in Cox's Bazar. The agenda of the meeting is enclosed at Annex B.

03. At the outset, Hon'ble Foreign Minister thanked the international community for their support to Bangladesh in providing humanitarian assistance to the Rohingyas. He hoped that the support would continue till the repatriation is completed. He stated that China is interested to help Bangladesh and Myanmar in the repatriation of the Rohingyas and accordingly organized the trilateral meeting among Bangladesh, Myanmar and China in Beijing in June 2018. He stated that as per his request made at the trilateral meeting, Myanmar government hosted Bangladesh delegation from 9-12 August 2018 in Myanmar. HFM then shared that the delegation which included members of the Joint Working Group (JWG) were allowed to visit Rakhine State where they witnessed the current state of preparation of return of the Rohingyas. He mentioned that among the countries who have committed for infrastructure development in Rakhine State, India has already started physical works with building houses in Maungdaw Township. He pointed out that Japan, China and Indonesia all are willing to work for infrastructure and socio-economic development of the Rakhine state. He also referred to his meetings with Japanese Foreign Minister and Japanese Ambassador in Myanmar where they informed Bangladesh that they advised Myanmar to close down the IDP camps and to allow the Rohingyas to move freely.

04. With regard to Myanmar's seriousness about repatriation, Hon'ble Foreign Minister stated that, Myanmar is under pressure from international community to address the issue of the Rohingyas, especially after the publication of report of the Independent International Fact Finding Mission on Myanmar. Referring to the recent incident where Myanmar Army had to publicly apologize for using photos of Bangladesh's Liberation War and Hutu Refugees of Rwanda in 1996 in their official photo book to falsely showcase 'atrocities of Rohingyas against Burmese people',

Hon'ble Foreign Minister mentioned that this kind of apology-seeking is unprecedented for them. He mentioned that, due to the increasing pressure regarding accountability, Myanmar is now showing willingness to start the repatriation and they would like to start repatriation with already verified 3,000 Rohingyas. He mentioned, Myanmar has also agreed to receive a Rohingya delegation from the camps in Cox's Bazar to their villages in Myanmar to see the situation by themselves. In this regard, Hon'ble Foreign Minister advised the members of the NTF to concentrate in the repatriation related work as per the agreements signed with Myanmar including building the transit camps and filling up the forms. He also sought support from UN agencies to help Bangladesh to begin repatriation process as soon as possible.

05. After Hon'ble Foreign Minister left the meeting, Foreign Secretary and the Chair of NTF apprised the meeting about Hon'ble Prime Minister's guidelines in the Rohingya issue. He informed the meeting that, according to the guidelines of HPM – i) NTF remains the top policy making body for all Rohingya related issues including humanitarian response; and ii) a 09-member 'Repatriation Taskforce' chaired by RRRC will coordinate the repartition related activities. Foreign Secretary also informed the meeting that as articulated by HFM, repatriation of 3000 verified Rohingyas is our priority now. The Repatriation task force should immediately begin its work. If the Rohingya, already verified by Myanmar wants to go back to Myanmar, they should be allowed; if they don't, that decision too should be transparent and on record. UNHCR was advised to take necessary actions in this regard including conducting intention survey among the verified Rohingyas in consultation with the government.

06. The Chair then invited the World Bank to update on their emergency project assistance for the Rohingyas. The World Bank representative mentioned that the World Bank has agreed to financing from the IDA 18 refugee sub-window on grant terms on an exceptional basis to support the refugees. For any project, 5/6 of the financing will come from the sub-window and 1/6 will come from the Bangladesh's core country IDA allocation which is in credit terms. To enable Bangladesh to access 100% grant financing to support the refugees, the World Bank is currently consulting with development partners for buying down the remaining the 1/6 credit financing. Support to the host communities will be provided through existing operations financed by Bangladesh's core country IDA allocation. The Chair intervened saying that a high level Bangladesh delegation would attend the upcoming UN General Assembly in New York and there might be scope for discussing with the friendly countries regarding buying down the rest 1/6 (GoB portion) of the WB finance. World Bank representative appreciated the idea.

07. The World Bank representative stated that based on consultation with various stakeholders in Cox's Bazar and Dhaka and Bank's needs and vulnerability assessment, the initial program will include investment financing and technical assistance to support government priorities and complement humanitarian efforts in

03 areas (USD 240 million in total), which are- i) USD 50 million for health, nutrition, population; (already approved in June 2018), ii) USD 20 million for education; and, iii) USD 165 for multi-sectoral project. Regarding education, the World Bank representative mentioned that they would only consider learning center based informal education for Rohingya children and tracking strictly based on NTF guideline (informal, no skill training, no Bangla education). The Chair reiterated that government policy for education to the Rohingyas has to be followed. On a query from Director General (UN) the World Bank representative clarified that the renovation of local schools is basically meant for the host community as those structures have suffered damages during early days of the influx. These renovating of the local school do not imply that these will be used for the Rohingyas. Rather, the education of the Rohingyas will take place in the learning centers inside the Camps.

08. The ADB representative informed that the ADB Board has already approved USD 100 million. The project will be executed by 4 agencies, LGED (USD 30 million), DPHE (USD 35 million), RHD (USD 27 million), BREB (USD 8 million). He further mentioned that they are in different stages of contract awarding. He appreciated NTF for leading the coordination mechanism which has been very useful for them. He requested for early approval of establishing an ADB site office at the premise of UNHCR Office in Cox's Bazar. ADB also informed that the ground level coordination and cooperation with the UN agencies is running quite well. In this regard they referred to their recent project to build sheds for WFP's food distribution.

09. The UN Resident Coordinator informed that field level coordination for humanitarian assistance has been good so far. But, the World Bank and the ADB need to have more conversations with the existing coordination mechanism led by UN system at the upper level. RRRC proposed to include one representative from the ADB and the World Bank each in the local level coordination mechanism for humanitarian response. The Chair advised the World Bank to join the existing coordination framework both at the ground level and at the central level. He also requested UNRC to convene a meeting with the country representatives of World Bank and ADB. In terms of implementation of projects, the Chair advised WB demonstrate flexibility as the area of operation and the nature of the projects are completely different from WB's usual projects.

10. The UNRC raised the issue of humanitarian assistance post December 2018 and proposed to develop a medium-term response plan for further one more year. The Chair advised that the new plan may be developed for six months in line with the existing one. The Chair also asked the UNRC to provide a list of priority areas where additional funding is required so that the VVIP delegation can solicit support from possible donors during the upcoming UNGA in New York.

11. The Project Director of Bhasan Char development project informed that the embankments construction has been completed and only 23% of the overall work is

left. The relocation of the Rohingyas to Bhasan Char would start in early October with 60 families. UNHCR country representative emphasized that there should be a technical assessment of the area before relocation could begin and UNHCR has the expertise to help Government of Bangladesh in this regard.

12. UNHCR representative Mr. James Lynch updated about the development related to MoU signed between the UNHCR and Myanmar government. He informed that an opportunity has arisen for the Rohingyas living in the zero line to go back to their homes in the Rakhine State. Pointing on the Myanmar Minister's claim during the Bali Ministerial Conference that Rohingyas were not forced to leave rather they fled Myanmar for taking the opportunity of 3rd country resettlement, Mr. Lynch questioned the political intention of the Myanmar government. Regarding the practical difficulties in returning the verified Rohingyas immediately, UNHCR representative said that some of the verified Rohingyas have been termed as terrorists by Myanmar and there are people whose family members have not been verified. The Chair mentioned that Myanmar has accused Bangladesh of not cooperating in the repatriation of Rohingyas and they are running propaganda saying that the government of Bangladesh is not willing to return the Rohingyas. In the face of such falsehood, the government has decided to start the repatriation process and that would be done with full respect to relevant international laws. If the verified Rohingyas decline to return, that would be put on record for information of the international community.

13. Additional Secretary, Public Security Division mentioned that security in the camps area becomes vulnerable after the dusk due to poor accessibility and poor lighting. He requested the donor agencies to consider putting additional lighting in the camps area, improving road networks inside, building watch towers for the LEAs etc. The representative from the Ministry of Fisheries and Livestock, who recently worked as a Camp In-charge, echoed the same concerns and added that after 6 pm host community and the Rohingya people get involved in illegal activities. He pointed that there should be police camps inside the Rohingya camps. The Chair invited the World Bank and the ADB to consider putting additional street lights/flood lights in the camps area urgently under the ongoing projects. It was known that WB is putting up 50,000 lights on a priority basis and ADB would put up 2000 lights in the camps. The Chair requested to scale up their services in this area. The Chair also informed the meeting that, Police would increase vigilance in the camps and there would be night patrols to curb crimes at night. The UNRC welcomed it saying such actions would help address the protection of the Rohingyas particularly of women and children.

14. The DG (UN) addressed the NTF and informed the meeting that UN Security Council had an open session on Rohingya issue on 28 August 2018. In that session, UNSG, PR of Bangladesh and Myanmar and UNHCR Goodwill Ambassador Cate Blanchett were present. In that session there was intensive discussion on Rohingya crisis. Bangladesh stated the cause of this manmade crisis and suggested the ways

forward to solve the crisis; whereas, Myanmar denied the responsibilities. DG (UN) further mentioned about the report of IIFFM published on 27 August 2018, where, Myanmar military and Government was held accountable for genocide, war crimes and crimes against humanity. The members of the IIFFM, established through UN Human Rights Council resolution, visited Bangladesh twice to collect data. They also used various other sources to get information. DG (UN) further mentioned that Hon'ble Foreign Minister participated in the Ministerial on Advancing Ministerial Freedom on 25-26 July 2018 at Washington DC which was arranged by USDOS. In that meeting, HFM described about the sufferings of the Rohingya people and the atrocities they faced. A number of statements were produced and adopted in that ministerial meeting, in particular a statement on the situation of religious freedom in Myanmar, which was co-signed by Bangladesh.

15. Regarding health issues, representative from the Ministry of Fisheries and Livestock (former camp in-charge) added his concerns for high fertility growth rate among the Rohingyas. Sharing the concern, the Civil Surgeon of Cox's Bazar mentioned that Rohingyas did not have knowledge or access to family planning methods and they often refuse to use contraceptives. However, the injectable contraceptives and the implants which are well accepted among the Rohingyas are facing shortage of supply. He also mentioned that, the total fertility rate could be decreased if the Rohingyas could have access to the permanent methods of family planning. But, the imprest money provided for this method is only for Bangladeshi nationals. He suggested M/o Health may be requested to withdraw the restriction. He also mentioned that till now 266 HIV positive cases have been identified and as Myanmar has a higher prevalence of HIV (0.8% compare to 0.01% in Bangladesh) there is risk that this would impact Bangladesh's health situation. What is even more alarming is that there is ongoing horizontal (people to people) and vertical (mother to baby) transmission of HIV which will increase the number of total cases. The children born with HIV are vulnerable and have many health complications.

16. Representative from Health Services Division of MoHFW mentioned about the communicable diseases as TB and Polio. Government of Bangladesh eradicated polio from Bangladesh, but, there have been cases of wild polio virus identified among Rohingyas. Government of Bangladesh is trying to combat the infectious diseases through Extended Programme on Immunization (EPI). But the existing cases are of great concern. Civil Surgeon of Cox's Bazar mentioned that so far 1532 cases of Tuberculosis has been identified. They are given the anti-TB treatment. The drugs / medicines needed for the treatment are met from the allocated medicines for Bangladeshi nationals in that area. It will be helpful if these medicines can be supplied in larger amount. As Hepatitis B is a highly contagious disease, it should be prevented through BCG vaccination and treated effectively. However, the severely malnourished Rohingyas in a crowded camp are the most vulnerable group for tuberculosis. The Chair said the UN agencies would be requested to scale up their assistance in public health. He also advised the health officials to address these resource gaps while finalizing the WB and ADB funded projects in health sector.

17. Regarding the issue of water supply and environment, representative from the Ministry of Fisheries and Livestock mentioned that there is no hydrologist in the camps. The environment is continuously deteriorating. If there is digging for water, there is presence of salinity, arsenic and heavy metals. He suggested that there should be a hydrologist to find out a sustainable solution to this problem. He also suggested that water reservoirs could be made to ensure access to fresh water.

18. Regarding the N visa issue of INGO officials, representative from NGO Affairs Bureau stated that currently 145 NGOs are working in the Rohingya camps. Among them 100 NGOs are National and 45 are INGOs. 41 NGOs have been blacklisted recently. The Chair suggested that the visa rules for the NGO workers should be followed strictly and no one should be allowed to work without proper visa.

19. In course of discussions, the meeting took the following decisions:

a) As approved by the HPM, NTF will continue to provide policy guidelines on Rohingya issue and the newly formed 09-member 'Repatriation Taskforce' led by RRRC will take action commence repatriation of the Rohingyas to Myanmar with priority. The members of the Repatriation Taskforce is attached at Flag-'B'.

b) Repatriation of nearly 3000 verified Rohingyas should start immediately. The office of the Repatriation Task force would be set up in Cox's Bazar and start immediate action to repatriate the verified Rohingya.

c) UNHCR would conduct "intention survey" of the verified Rohingyas after due consultation with the NTF and other govt. bodies.

d) The WB and ADB would be included in the existing UN coordination mechanism led by UNRC, UNHCR and IOM. At the local level, one representative from the ADB and the World Bank each would be included in the ISCG.

e) The World Bank would diligently follow the government's policy regarding learning facilities/education to the Rohingyas, which will be informal, without any skill training and without Bangla curriculum).

f) UNRC will send a list of priority areas that require further funding to the Ministry of Foreign Affairs.

g) The ADB would send a letter proposing establishment of a temporary site office in Cox's Bazar in the UNHCR premises.

h) The World Bank and the ADB would include putting additional lighting in the camps area and improving accessibility inside the Camps in their upcoming projects and implement these components on a priority basis.

i) Visa requirements of INGO workers would be strictly monitored and no one would be allowed to work without proper "N" visa. Airport Immigration should be advised to ensure compliance.

j) New Joint Response Plan would be developed in line with the existing one for 06 months (Jan - Jun 2019).

k) The Strategic Executive Group co-chairs (UNRC, IOM and UNHCR) would convene a coordination meeting with WB and ADB for discussion on needs and coordinate the responses.

l) UN system would reduce international staffs and appoint more Bangladesh Nationals.

m) The vehicles imported by the UN agencies will not be used by other non-UN entities and individuals. The UN Agencies will focus more on health materials, like medicines, vaccination, supplies for birth control, rather than capacity building in terms of logistics.

n) The health service providers would be requested to provide adequate family planning related supplies for the Rohingyas.

o) Ministry of Health and Family Welfare would propose to the World Bank and the ADB for including emergency supply for family planning and medicines for other contagious diseases.

p) MoFA will continue its diplomatic efforts to ensure safe, dignified and sustainable return of all forcibly displaced Rohingyas to their homeland in Myanmar.

20. With no other issues to be discussed, the Chair declared the meeting closed.

(Md. Shahidul Haque) 11/09/2019

Foreign Secretary (Senior Secretary)
& Chair, National Task Force on Implementation of National Strategy on Myanmar Refugees and Undocumented Myanmar Nationals

Minutes of the Twenty-Third Meeting of the National Taskforce on implementation of the National Strategy on Myanmar Refugees and Undocumented Myanmar Nationals (UMNs)

The twenty-third meeting of the National Task Force (NTF) on Implementation of the National strategy on Myanmar Refugees and Undocumented Myanmar Nationals (UMNs) was held on 22 November 2018 at the Ministry of Foreign Affairs. Mr. Md. Shahidul Haque, Foreign Secretary and the Chair of the NTF presided over the meeting. The list of participants at the meeting is furnished at **Annex-A**.

02. The agenda of the meeting included the recent efforts and progress made in repatriation, current situation with humanitarian response, and overall law and order situation in Cox's Bazar (**Annex B**).

03. The Chair in his welcome remarks said that the purpose of the meeting is to discuss the issues involving the planned repatriation on 15 November 2018 of the Forcibly Displaced Myanmar Nationals (Rohingyas) back to their homeland in Myanmar including evaluation of the roles played by various stakeholders in the process. He then invited the United Nations Resident Coordinator (UNRC) in Dhaka for updates from the UN Agencies in this regard.

04. UNRC, at the outset, recalled Bangladesh's sustained commitment to voluntary repatriation and thanked the Government of Bangladesh (GoB) for not repatriating the Rohingyas back to Myanmar against their will. As regards the condition in Myanmar, UNRC informed that the United Nations has intensified its efforts and working closely with the Government of Myanmar in creating conducive environment in Myanmar and thus to facilitate the voluntary return of the Rohingyas. She mentioned that National Taskforce is particularly of interest to them for seeking guidance on managing various kinds of risks associated with the situation in the Rohingya camps. She then invited Representative of UNHCR to update on repatriation process.

05. UNHCR representative referred to GoB's commitment to voluntary repatriation and appreciated the GoB for not forcing the Rohingyas to return to Myanmar. He mentioned that UNHCR made every effort to assess voluntariness; however was successful to reach only 6% (50 individuals) among the list of 2260 individuals verified by Myanmar for repatriation. He pointed out that safety and security in Myanmar couldn't be ascertained by UNHCR under the current situation although it remains a priority for UNHCR. To promote voluntary repatriation, UNHCR representative proposed to arrange a "go and see" visit for a delegation of Rohingya people from the camps in Bangladesh who would see for themselves whether the conditions are improving on the ground and report back to the vast majority of the Rohingyas. He offered to discuss the modalities for such arrangement. He also solicited commitment from Bangladesh that those going to Myanmar under 'go and see' visit would be allowed to return to Bangladesh.

06. The Chair expressed his dissatisfaction at the narrative of UN regarding possible 'forced return' and asked them to explain where did the Government of Bangladesh

suggested that the Rohingyas would be returned by force. He particularly reminded them of Hon'ble Prime Minister's statements and other references regarding GoB's commitment for safe, voluntary and dignified return. He referred to UNHCR's statement and press release prior to the planned repatriation, which were presumptive and focused only on Bangladesh. He inquired if there has been any public statement by UNHCR focusing on the condition in Myanmar including the lack of access to the villages by the United Nations even after having an agreement between Government of Myanmar, and UNHCR and UNDP. In response the UNRC updated the meeting that the UNDP Director is scheduled to visit Myanmar soon and UN's 3rd phase of assessment in the villages in Rakhine State will start soon.

07. The Chair then invited the Refugee Relief and Repatriation Commissioner (RRRC) to brief the meeting about the planned repatriation exercise. RRRC informed that UNHCR did not allow any GOB representative to be present during their voluntariness exercise. There have been reports that Rohingyas were deliberately asked leading questions so that they do not choose to go back, he added. In one of the two camps, while speaking to the prospective returnees, references were made to the atrocities, which influenced their choice, he added. He further stated that questions of voluntariness exercise should have been discrete. If someone wants to go back; there should not be any further question. If someone does not want to go back, only then further questions should be asked to understand their reasons for not going back, which then can be worked out with all the parties. RRRC informed that after hearing from various sources that Rohingyas had been asked such questions as "how many people of your family did get killed?" in the voluntariness exercise, he raised the issue with the concerned UNHCR staff who had been rude in his response.

08. The DGFI representative referred to the report of the RRRC and said that, they also have information that no GoB official was allowed to take part in the UNHCR's voluntariness exercise, despite Bangladesh's repeated assurance of not using any force in repatriation. He also raised the issue of asking leading questions to the prospective returnees in the voluntariness interview, as reported by many. He inquired to the UNHCR if there is any objective criterion to assess security condition, when they mean security condition is not conducive in Myanmar. Secretary (MAU), MoFA added in this regard that there has been no report of violence for quite some time in the Rakhine State of Myanmar.

09. The Chair expressed serious concern to such behavior of UNHCR and stated that GoB is appalled to know that no representative from the Government was allowed during the voluntariness assessment exercise. The Chair also invited an explanation from UNHCR about the allegations raised against its staffs. He mentioned that the UNHCR should investigate the issue and proper action should be taken against the concerned UNHCR official, if the allegations of misconduct are found true. The meeting decided that UNHCR would provide a report on the recent attempt of repatriation where it would respond to the allegations raised in the meeting. In future, UNHCR officials have to maintain professionalism and talk responsibly specially to the media, which has been grossly disregarded in the recent case. It was also decided that at least one representative from GoB, (RRRC) will be present in any future voluntariness exercise and the questionnaire would be developed by the National Task Force.

Humanitarian Assistance/JRP:

10. IOM representative informed that the Second Joint Response Plan (JRP) for 2019 is under drafting phase. The Plan would be worth USD 859 million this time. There will be sufficient focus on localization of humanitarian response with support from the GoB and it will prioritize on lifesaving, keeping in mind the environmental degradation, and impacts on the livelihoods of the host community and how to mitigate this. IOM representative further added that protection and gender mainstreaming would be key factors in the response with the focus on both the Rohingyas and the host communities. Coordination mechanism will be further strengthened – bringing more and more government to the center of the structure, with more responsibilities given to the RRRC. IOM representative stated that ISCG would also focus on localization of staff for more effective implementation.

11. Regarding inclusion of anti trafficking and anti radicalization measures in the new JRP, the meeting was informed that trafficking would fall under the protection part of the response, which is being taken care of by UNHCR and UNICEF. In addition, IOM also briefed about its anti-trafficking initiatives in Cox's bazar district, which would include the Rohingyas. Regarding the PVE/ anti-radicalization measures, UNRC informed that there is no specific programme to address the radicalization; however UN's programme on 'social cohesion' for the entire Cox's Bazar district would address it. She also stated that the issue would be further discussed with MoFA.

12. The representatives from Public Security Division stated that according to the bilateral arrangement of return the repatriation should be completed within 02 years. Therefore, the humanitarian actors should develop their projects for short term only. The representative from Police Headquarters raised the issue of growing dissatisfaction among the host community due to competition over land, employment opportunities, though they were very welcoming initially on humanitarian consideration. The Rohingyas are becoming increasingly involved in crimes and so far 490 Rohingya have been found involved in major crimes including murder, rape and domestic violence. Protracted presence of the Rohingyas in Bangladesh will eventually turn the host community hostile. RRRC said that demographic imbalance is another source of frustration therefore; there should be enough allocation of resources to the host community. The representative from DGFI stated that radicalization risk would increase if the repatriation were delayed.

13. UNRC stated that frustration among the Rohingyas increases the risk of radicalization. She highlighted on guidelines to minimize frustration. About localization of UN staffs, she mentioned that UN is taking it seriously. However, it would take some time. About JRP, UNRC mentioned that JRP has to be need-based. The Chair urged to keep the needs of the host community in consideration. The Chair stated that the next JRP must continue to remain temporary in nature. He requested to share the draft of the JRP immediately as the Government will carefully review the draft.

14. The DGFI representative informed that World Food Programme (WFP) has been found flying drones above the camps and well beyond the camps without any prior approval of the Government authorities. There was also a complaint of forceful acquisition of land by

WFP from a private individual, informed the representative of AFD. The Chair expressed dissatisfaction over flying drones without government approval and forceful acquisition of land. He mentioned that any request for use of drones or telecommunication equipment must go through the Ministry of Foreign Affairs. He also requested UNRC to look into the matter very seriously and report back to the Chair of the NTF.

15. The Chair raised the issue of wide-spread abuse of visa by the humanitarian workers. Any further misuse will be treated strictly, he added. He also added that the Government has decided to end the flexible provision (under emergency situation after 25 August 2017) of issuance of visas to the humanitarian workers, media representatives etc. The Chair mentioned that from now on visa application has to be submitted at least 2 weeks before the intended travel date and the Missions will issue proper visa based on documents submitted along with the visa application. In this regard, Airport Immigration would be advised to ensure compliance so that no one can enter with wrong visa category as they are supposed to obtain special visa for visiting the Rohingyas Camp as decided earlier. On request of UNRC for organizing a working level meeting regarding visa issues, the Chair asked UN Wing MOFA to facilitate setting up a meeting with the relevant GoB stakeholders.

16. The Chair mentioned that the presence of Rohingyas have created multiple burdens on the MoFA, PMO, MoHA, MoDMR, law enforcement agencies etc. He highlighted the excessive visits to Cox's Bazar by the donors, humanitarian actors, embassy officials, media etc, which is putting additional burden, especially on the local law enforcement agencies, the local administration including RRRC and DC. He referred to the upcoming general election for which most of the security agencies will remain busy, and stated that it would become difficult for the government to provide police escorts and other security facilities to the dignitaries. He requested humanitarian actors to take these factors into account and limit the high level visits to Cox's Bazar for now. He also reminded that the organizers of such visit must keep the Foreign Office informed well ahead of time, as it requires much time to obtain security approval.

17. The issue of cash distribution for winter was raised and the RRRC requested whether the Rohingyas could be provided with limited amount of cash for buying winter clothes and blankets from the local market as per their needs. The Chair mentioned that no cash should be distributed to the Rohingyas; rather some cash can be topped up in cards to the Rohingyas for buying winter clothes of their choice.

18. Representative from DGFI highlighted the decision taken earlier by the NTF that No permanent structure will be built in the camp for long-term use, so that it does not generate impression that Rohingyas are going to stay Bangladesh for long time. The RRRC mentioned that usually they don't allow any permanent structure within the camps but they had to build some structures using building materials considering the purpose and the nature of those establishments, such as offices of the camps-in-charge and the WFP food storage. The Chair stated from his previous experience in working with refugee conditions such construction of permanent structure was not necessary as modern tents and pre-fabricated temporary structures are available and used globally for various purposes in humanitarian operations. The Chair advised RRRC not to issue any permit without consulting NTF and also advised

him to remain vigilant so there is no permanent structure built in the camp sites without clearance from NTF.

19. There was further discussion on security measures in the Rohingya camps in the run up of the national election. Representative from the Cox's Bazar DC Office mentioned that due to the upcoming National Parliament Election duty, the DC Office would remain extremely occupied to ensure the necessary arrangements for the election. They have already requested for additional seven platoons of security forces in the Cox's Bazar District. In such situation, it would be difficult for the local administration to provide extra security/police escorts for the foreigners separately to visit the camps. Hence, during the time of national parliamentary elections, frequent visit by the INGOs and dignitaries to the Camps should be discouraged unless they have any strong reason to visit the camp urgently. Representative from the BGB mentioned that during election time, Rohingya camps visit should be restricted and any kind of visit should not be allowed at least from seven days before the Election date. RRRC requested that the police camps inside the camps should be brought under the camp-in- charge. The Chair stated that this would require consultation with the IGP.

20. Representative from AFD stated that the transit camps prepared for the repatriation should be kept secured and may be fenced for security reasons. He also requested for installation of 02 fire brigades for the camp areas. He also referred to the frequent and unabated use of 4G networks in the camp area and stated that BTRC should be requested to bring the network in that area to 2G. Representative from the Coast Guard highlighted the issue of installing watchtower as it has become difficult for the coast guard to monitor the movements of boats, which are used for trafficking. He requested that all LEAs should exchange intelligence among themselves and IOM may be requested to support activities in preventing and countering human trafficking. Referring to the security issues during the election period, the Chair advised the security forces to ensure highest level of security measures for the Rohingya Camps.

21. As regards the repatriation of the Rohingyas, the meeting discussed about stronger diplomatic efforts and engagement of UN is a positive manner. The RRRC stated that the Rohingyas would be encouraged to return is Myanmar could assure them that they would be offered pathway to citizenship and they will not end up living in the camps. The Chair stated that MoFA is considering various diplomatic options for early and sustainable return of the Rohingyas and are already engaged with UN, friendly countries and regional organizations in this regard.

22. In course of discussions, the meeting took the following decisions:

a) UNHCR will provide a report on its role in the recent repatriation on 15 November 2018 and also clarify its position on situation in Myanmar.
b) UNHCR will conduct investigation on the allegations of misbehavior of its staffs with RRRC and ensure that its officials maintain professionalism in their conduct and talk responsibly, specially to the media.

c) NTF will develop a new questionnaire for voluntariness exercise. There would not be any leading question and if someone wants to go back, no further question would be asked.
d) UNHCR will ensure that one representative from GoB (RRRC) will be present in any future voluntariness exercise.
e) The new JRP would be shared with MoFA at the earliest possible time for review of the NTF.
f) The JRP should be temporary in nature and may include anti-trafficking and anti-radicalization measures.
g) No one should use drones or any kind of telecommunications equipment without prior approval from the GoB and all such requests must channel through MoFA.
h) The UN staffs, INGO workers, Press and all humanitarian actors must apply for visa through Bangladesh missions abroad with supporting documents and come to Bangladesh with proper visa. SSD of MoHA would be requested to take measures in this regard.
i) Immigration officials would stop any visitors arriving in Bangladesh to work in Cox's Bazar without appropriate visas.
j) Visit by foreign dignitaries and donors to the Rohingya camps would be limited for the time being and the visits should be facilitated by ISCG only after receiving clearance from MoFA.
k) The Chair mentioned that no cash should be distributed to the Rohingyas; rather some cash can be topped up in cards to the Rohingyas for buying winter clothes of their choice.
l) No permanent structure would be made in camps without prior approval of the NTF.
m) Security forces will ensure highest level of security measures for the Rohingya Camps.
n) MoFA will continue its diplomatic efforts to ensure sustainable return of the Rohingyas with support from international community.

23. With no other issues to be discussed, the Chair declared the meeting closed.

(Md. Shahidul Haque)

Foreign Secretary (Senior Secretary) and Chair, National Task Force on Implementation of the National Strategy on Myanmar Refugees and Undocumented Myanmar Nationals
