# Supplementary material for: Challenges of healthcare financing in the world’s largest refugee camp: a mixed-method study among healthcare stakeholders for Rohingya refugees in Bangladesh
Source: BMJ Open. 2025 Jan 23;15(1):e083021. doi: 10.1136/bmjopen-2023-083021 (PMC11784382; doi:10.1136/bmjopen-2023-083021)
Supplement: Supplementary file 4 [file bmjopen-2023-083021.reviewer_comments.pdf]

PEER REVIEW HISTORY

BMJ Open publishes all reviews undertaken for accepted manuscripts. Reviewers are asked to complete a checklist review form and are provided with free text boxes to elaborate on their assessment. These free text comments are reproduced below.

ARTICLE DETAILS

Title (Provisional)

The challenges of healthcare financing in the world's largest refugee camp: A mixed-method study among healthcare stakeholders for Rohingya refugees in Bangladesh

Authors

Talukdar, Imdadul Haque Haque; Tarannum, Sayeeda; Smith, Isabel; Rifat, M. A.; Boga, Priyanka; Mumin, Jubayer; Veronés, Pablo André; Mahdi, Ziba; Shoshi, Saima Alam; Khan, Arif Faysal

---

VERSION 1 - REVIEW

**Reviewer**	**1**
**Name**	**Bakhtiari , Ahad**
**Affiliation**	**Tehran University of Medical Sciences**
**Date**	**25-Dec-2023**
**COI**	**No**

---

1. Please mention in the introduction and method and findings the approach that provides services to immigrants, these models are:

Various countries have developed four care models to serve refugee and migrant populations, including mainstream services, specialized-focus services, gateway services, and limited services.

Please see:

Mapping health systems' responsiveness to refugee and migrant health needs. Geneva: World Health Organization; 2021. Licence: CC BY-NC-SA 3.0 IGO.

2. In the introduction, explain more about the characteristics of one million refugees.

Age group, literacy level, cultural and linguistic difference, social and economic status

3.In theme one, if you have more findings regarding pooling and allocation for humanitarian assistance, please explain more. What are these processes and what are their strengths and weaknesses?

4.In the end, if you have a proposed policy option that can improve the health status of refugees in your country, present it in the form of policy options.

**Reviewer**	**2**
**Name**	**Abidi, Saba**
**Affiliation**	**Delhi Pharmaceutical Sciences and Research University**
**Date**	**07-Jan-2024**
**COI**	**no competing interests**

Methods section in the abstract could be more elaborate.

More targeted specific outcomes need to be mentioned

The semi structured questionnaire should be attached in supplementary material.

A more detailed explanation on how the biases were taken care of.

limitations not mentioed

**Reviewer**	**3**
**Name**	**Tarawneh, Mohammad Rassoul**
**Affiliation**	**International Academy of Public Health**
**Date**	**26-Jun-2024**
**COI**	**No conflict of interests**

No comments

VERSION 1 - AUTHOR RESPONSE

**Reviewer-01: Dr. Ahad Bakhtiari , Tehran University of Medical Sciences**	
**Reviewer's Comment**	**Author's Response**
1. Please mention in the introduction and method and findings the approach that provides services to immigrants, these models are:<br>Various countries have developed four care models to serve refugee and migrant populations, including mainstream services, specialized-focus services, gateway services, and limited services.<br>Please see:<br>Mapping health systems' responsiveness to refugee and migrant health needs. Geneva: World Health Organization; 2021. Licence: CC BY-NC-SA 3.0 IGO.	Thank you for this valuable comment. We have mentioned the approach that provides services to immigrants. Different healthcare models were added to the introduction, methods. However, we could not adjust the changes in the findings section. As we worked according to the objective of the study. The four-care model was not aligned the findings. Please see the changes on **pages 4 and 5 lines 121-138**.

2. In the introduction, explain more about the characteristics of one million refugees. Age group, literacy level, cultural and linguistic difference, social and economic status	We have added more about the characteristics of the one million refugees. For example, age group, literacy level, cultural and linguistic difference, and socio-economic status have been added. Please look at **pages 3 and 4, Lines 109-116**.
3. In theme one, if you have more findings regarding pooling and allocation for humanitarian assistance, please explain more. What are these processes and what are their strengths and weaknesses?	In theme-01, the issue of pooling and allocation of humanitarian assistance with its strengths and limitations have been included. Please investigate the **pages 13-14 and lines 323-337.**
4. In the end, if you have a proposed policy option that can improve the health status of refugees in your country, present it in the form of policy options.	In the discussion part, we have proposed a few policy options based on our study findings which are expected to improve the health status of the Rohingya refugee in Bangladesh. Please look on **page 22, lines 518-530.**
**Reviewer-2: Dr. Saba Abidi, Delhi Pharmaceutical Sciences and Research University**	
**Reviewer's Comment**	**Author's Response**
1. Methods section in the abstract could be more elaborate.	Thank you for your comment. We have added more information in the methods section. The abstract has been modified. **Please see the changes on page 02 lines 51-79 and page 07 lines 189. On page 09 lines 222-228**
2. More targeted specific outcomes need to be mentioned	We have added more targeted specific outcomes according to your comments. The change is visible **on page 07 line 183-185.**
3. The semi structured questionnaire should be attached in supplementary material.	The semi-structured interview questionnaire has been added as supplementary file 02 in the list of supplementary files.
4. A more detailed explanation on how the biases were taken care of.	A new section has been inserted on how the biases were controlled **on page 11 and lines 264-276.**
5. limitations not mentioned	Thank you for your comment. We have added the limitations section **on page 2 and 3 lines 89-92.**

VERSION 2 - AUTHOR RESPONSE

**Reviewer-01: Dr. Ahad Bakhtiari , Tehran University of Medical Sciences**	
**Reviewer's Comment**	**Author's Response**

5. Please mention in the introduction and method and findings the approach that provides services to immigrants, these models are:<br>Various countries have developed four care models to serve refugee and migrant populations, including mainstream services, specialized-focus services, gateway services, and limited services.<br>Please see:<br>Mapping health systems' responsiveness to refugee and migrant health needs. Geneva: World Health Organization; 2021. Licence: CC BY-NC-SA 3.0 IGO.	Thank you for this valuable comment. We have mentioned the approach that provides services to immigrants. Different healthcare models were added to the introduction, methods. However, we could not adjust the changes in the findings section. As we worked according to the objective of the study. The four-care model was not aligned the findings. Please see the changes on **page 5 lines 99-117 in the highlighted version and in the page 5, lines 97-113 in the cleaned version**.
6. In the introduction, explain more about the characteristics of one million refugees.<br>Age group, literacy level, cultural and linguistic difference, social and economic status	We have added more about the characteristics of the one million refugees. For example, age group, literacy level, cultural and linguistic difference, and socio-economic status have been added. Please look at **page 4, lines 87-94 in the highlighted version and page 4, lines 85-92 in the cleaned version**.
7. In theme one, if you have more findings regarding pooling and allocation for humanitarian assistance, please explain more. What are these processes and what are their strengths and weaknesses?	In theme-01, the issue of pooling and allocation of humanitarian assistance with its strengths and limitations have been included. Please investigate the **pages 15 and lines 298-312 in the highlighted version and page 15, lines 295-309 in the cleaned version.**
8. In the end, if you have a proposed policy option that can improve the health status of refugees in your country, present it in the form of policy options.	In the discussion part, we have proposed a few policy options based on our study findings which are expected to improve the health status of the Rohingya refugee in Bangladesh. Please look on **page 24, lines 493-505 in the highlighted version AND page 24, lines 489-501 in the cleaned version.**
**Reviewer-2: Dr. Saba Abidi, Delhi Pharmaceutical Sciences and Research University**	
**Reviewer's Comment**	**Author's Response**
6. Methods section in the abstract could be more elaborate.	Thank you for your comment. We have added more information in the methods section. The abstract has been modified. Please look into the **pages 02-03 lines 26-53 (both in marked-up and cleaned versions).**
7. More targeted specific outcomes need to be mentioned	We have addressed this comment more elaborately in the end of this table.
8. The semi structured questionnaire should be attached in supplementary material.	The semi-structured interview questionnaire has been added as supplementary file 02 in the list of supplementary files.
9. A more detailed explanation on how the biases were taken care of.	A new section has been inserted on how the biases were controlled **on page 12 and lines 242-254 in the marked-up**

	**version AND pages 12-13, lines 240-252 in the cleaned version.**
10. limitations not mentioned	Thank you for your comment. We have added the limitations in the form of key points in the page 3, lines 63-70 (marked-up version) AND page 3, lines 63-68 (cleaned-version).<br><br>Moreover, a separate sub-section titled “strengths and limitations” has been inserted in the pages 24-25, lines 506-524 both in the marked-up and 502-520 cleaned versions.
